# Molecular Dynamics Reveal Binding Mode of Glutathionylspermidine by Trypanothione Synthetase

**DOI:** 10.1371/journal.pone.0056788

**Published:** 2013-02-25

**Authors:** Oliver Koch, Daniel Cappel, Monika Nocker, Timo Jäger, Leopold Flohé, Christoph A. Sotriffer, Paul M. Selzer

**Affiliations:** 1 MSD Animal Health Innovation GmbH, Schwabenheim, Germany; 2 MOLISA GmbH, Magdeburg, Germany; 3 Institute of Pharmacy and Food Chemistry, University of Würzburg, Würzburg, Germany; 4 Otto-von-Guericke-Universität, Magdeburg, Germany; 5 Interfaculty Institute of Biochemistry, University of Tübingen, Tübingen, Germany; 6 Wellcome Trust Centre for Molecular Parasitology and Division of Infection & Immunity, Faculty of Biomedical & Life Sciences, University of Glasgow, Glasgow, United Kingdom; Technion-Israel Institute of Technology, Israel

## Abstract

The trypanothione synthetase (TryS) catalyses the two-step biosynthesis of trypanothione from spermidine and glutathione and is an attractive new drug target for the development of trypanocidal and antileishmanial drugs, especially since the structural information of TryS from *Leishmania major* has become available. Unfortunately, the TryS structure was solved without any of the substrates and lacks loop regions that are mechanistically important. This contribution describes docking and molecular dynamics simulations that led to further insights into trypanothione biosynthesis and, in particular, explains the binding modes of substrates for the second catalytic step. The structural model essentially confirm previously proposed binding sites for glutathione, ATP and two Mg^2+^ ions, which appear identical for both catalytic steps. The analysis of an unsolved loop region near the proposed spermidine binding site revealed a new pocket that was demonstrated to bind glutathionylspermidine in an inverted orientation. For the second step of trypanothione synthesis glutathionylspermidine is bound in a way that preferentially allows N^1^-glutathionylation of N^8^-glutathionylspermidine, classifying N^8^-glutathionylspermidine as the favoured substrate. By inhibitor docking, the binding site for N^8^-glutathionylspermidine was characterised as druggable.

## Introduction

The protozoan parasites of the genera *Trypanosoma* and *Leishmania* cause neglected diseases such as Chagas’ disease, African sleeping sickness or the various forms of Leishmaniasis, which *in toto* still account for about 100 000 fatalities *per annum* [1] (http://www.who.int/mediacentre/factsheets/fs259/en/index.html). Current treatments of these diseases are unsatisfactory and the lack of economic incentives is still hampering the development of new drugs by the pharmaceutical industry [2], [3]. However, increasing understanding of the parasites’ biology, related genome analyses and drug design technologies have more recently revived the search for efficacious and safe trypanocidal drugs [4], [5]. The preferred strategy consists in the identification of metabolic pathways that are essential for parasite survival and absent or sufficiently different in their mammalian hosts to enable selective inhibition of suitable molecular targets. An attractive example is the trypanothione system which is only found in trypanosomes and other *Kinetoplastida* [6], but not in any mammalian species [7].

In trypanosomatids, trypanothione [T(SH)_2;_ N^1^,N^8^-bis(glutathionyl)spermidine] is the key redox metabolite that directly or indirectly provides the reduction equivalents for multiple pathways that in mammals depend on glutathione (GSH) or thioredoxins. In fact, the only enzymes that efficiently use GSH in trypanosomatids appear to be glutathionylspermidine synthetase (GspS) and trypanothione synthetase (TryS). As first discovered in *Crithidia fasciculate* [8], T(SH)_2_ is the proximal reductant for thioredoxin-related proteins called tryparedoxins, which in turn reduce a variety of tryparedoxin peroxidases, which may belong to the peroxiredoxin or the glutathione peroxidase family (reviewed in [9]), the ribonucleotide reductase [10] and proteins implicated in proliferative control [11]. Without the aid of tryparedoxin, T(SH)_2_ substitutes for GSH in S-transferase reactions and regenerates ascorbate as substrate for heme peroxidases in *T. cruzi* and *Leishmania* species. Most of the enzymes constituting this system have been demonstrated to be essential [12]–[15]. TryS, considering its low cellular abundance, uniqueness of sequence [16], druggability [13], [17], [18] and dominant role in trypanosomatid redox metabolism, is accepted as a most attractive molecular target to search for trypanocidal drugs [7], [17].

We therefore reasoned that molecular modelling and docking combined with molecular dynamics simulations might be the approaches of choice to further clarify the mode of substrate binding and mechanistic details of TryS. Here we present *Lm*TryS models which in essence confirm the presumed binding sites for all substrates and, in particular, reveal how the intermediate Gsp moves from the site of its formation into a position suitable for its glutathionylation. Moreover, the Gsp binding site is here characterized as druggable.

**Figure 1:**
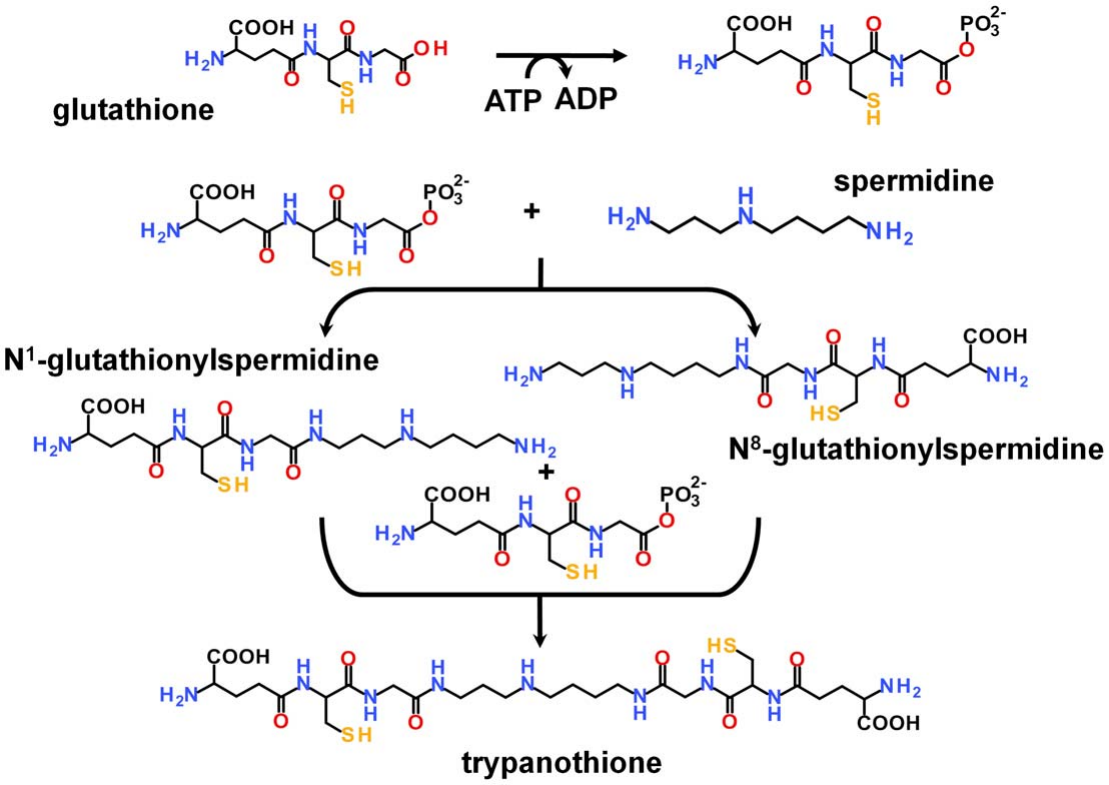
Two-step synthesis of trypanothione. For both steps, GSH has first to be activated by phosphorylation. Due to the asymmetry of spermidine the first step of the trypanothione biosynthesis could lead to two different glutathionylspermidines.

TryS is an unusual enzyme. It not only catalyses the synthesis of T(SH)_2_, but also its breakdown; the latter activity residing in a distinct domain [19]. But also the mechanism of T(SH)_2_ synthesis itself has for long remained enigmatic. Initially thought to require two distinct enzymes, GspS and TryS [20], TryS proved to be competent and sufficient to catalyse both steps of T(SH)_2_ synthesis [16], [21], [22]. This implies that GSH has to be conjugated to both of the rather remote terminal amino groups of spermidine (Sp) by a single enzyme (Fig. 1), which mechanistically is not easily understood. As discussed by Comini et al. [16], i) two distinct reaction centres could add GSH to N^1^ and N^8^ of Sp, ii) a single reaction centre could be flexible enough to carry the activated GSH to either of the Sp amino groups or iii) the intermediate Gsp has to change its binding mode to offer the second amino group for glutathionylation. With the structural elucidation of TryS of *L. major* (*Lm*TryS) by Fyfe et al. [19], the first possibility could be ruled out: The synthesis domain only contains a single binding site for ATP. The latter, like that of *Ec*GspS [23] and other ligases, displayed a typical ATP grasp fold into which ATP and two Mg^2+^ ions could be accommodated, although the loop presumed to firmly keep the ATP in place (residues 552 to 578) was not visible [19]. Further, a characteristic cleft to accommodate GSH in a way that its glycyl carboxylate could be phosphorylated was detected, while the putative Sp binding site appeared less structured. The triangular arrangement of these three putative binding sites appeared rather rigid and, in consequence, Fyfe et al. proposed a re-location of the intermediate Gsp as the only left alternative to synthesize T(SH)_2_. Unfortunately, however, the region where Gsp was supposed to bind (residues 251 to 261) also turned out to be disordered in the crystallographic analysis [19].

## Materials and Methods

### TryS Model Creation

During this computational analysis, four different TryS-models of increasing complexity were created and subjected to molecular dynamics simulations.

**Figure 2:**
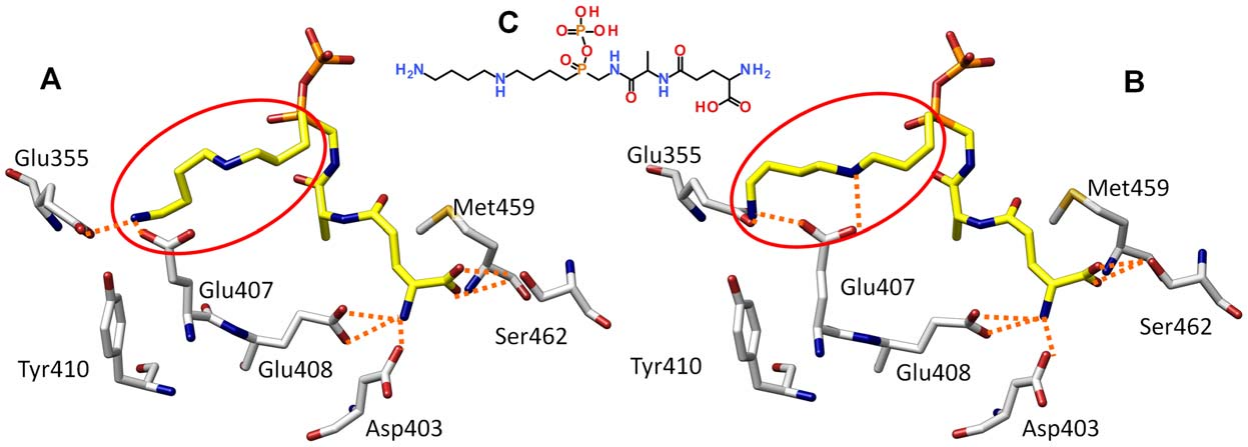
Binding modes of a Gsp homologue. Two major conformations of the spermidine moiety of the Gsp analogue (C) are observed. In the binding mode A loss of hydrogen bonds (orange dotted lines.) to Glu407 suggests weaker binding than in binding mode B.

The first *Lm*TryS model contains ADP and a Gsp analogue in which the carboxamide group that links GSH to spermidine is replaced by a phosphorylated phosphinate (see below, Fig. 2C). It is based on a crystal structure (pdb: 2ioa) of *E. coli* glutathionylspermidine synthetase (*Ec*GspS) [23] that contains these ligands. *Lm*TryS and *Ec*GspS have a sequence identity of ∼30% [19], and a structural overlay of the Cα-atoms of both synthetase domains is possible with an rmsd of 1.54 Å^19^. The synthetase domains of the enzymes were superposed by MOE [24] (see Figure S1 in Supporting Information S1 section I) and the Gsp analogue, ADP and both Mg^2+^ ions were transferred from the *Ec*GspS structure into the *Lm*TryS model and subjected to energy minimisation by the pertinent MOE function and MFF94x force field with relaxation of surrounding residues. Missing loop regions were added using the MOE’s homology modelling module. The best scored model (based on electrostatic solvation energy [25]) with both loop regions was used as starting conformation for molecular dynamics simulation.

The second *Lm*TryS model is based on a *Ec*GspS crystal structure (pdb: 2io7) that contains the ATP-analogue AMPPNP, thus indicating the position of the γ-phosphate of ATP [19]. After superposition as before, AMPPNP and the Mg^2+^ ions were transferred into the empty TryS crystal structure (pdb: 2vps). AMPPNP was modified into ATP by replacing NH by O. GSH was docked by GOLD [26] into its putative binding region corroborated by the MD simulation performed on model 1. Missing loop regions were added as above. The resulting model was subjected to MD simulation.

For the final models, *i. e.* those with all substrates bound, protein conformations from the previous MD simulation were used. A hierarchical clustering of the different protein conformations shows 5 clusters (see Figures S9 and S10 in Supporting Information S1 section III). Using the ensemble docking feature of Gold 5.0, both glutathionylspermidine molecules (N^1^-Gsp and N^8^-Gsp, see Fig. 1) were simultaneously docked into representative conformations of these clusters to identify the best combination. For the final models, the best scoring poses were used.

### Docking and Scoring

For docking GOLD 5.0 was used with standard settings and Goldscore for pose creation [26]. In each docking run, 100 poses were created and rescored using DrugscoreCSD [27]. The best scored pose was used for further analysis. If more than one protein structure was available, the ensemble docking feature [28] was applied to select the best combination of a protein conformation and a particular ligand pose according to maximum fitness value.

### MD Simulations

MD simulations were performed using the software package Amber in version 10 [29]. The protonation pattern of all His residues was checked with the Protonate3D function in MOE 2009 and assigned accordingly. Atomic charges for the ligands and the cofactor were determined using the RESP [30] methodology at the HF/6–31G* level in Gaussian 03 [31]. Missing force field parameters of the ligands were determined using the PARMCHK [32] module in AMBER according to the GAFF force field [33]. The missing force field parameters for ATP were taken from the AMBER parameter database [34]. The correct GAFF atom and bond types of the ligand were determined with the ANTECHAMBER program [32]. For the protein atoms the Parm99SB force field of AMBER [35] was used. The missing protons were added to the protein using the program LEaP. With this software, the starting coordinate and topology files for the simulations were built. Complexes were minimized with the module SANDER over 200 steps using the steepest descent algorithm and a modified generalized Born implicit solvent model [36]. Sodium ions were added to each of the minimized structures to obtain neutrality. Using LEaP the complexes were then solvated with the TIP3P solvent model [37] in a box the borders of which are at least 8.0 Å away from the protein, which resulted in the addition of about 25000 water molecules to the system. The system was then heated from 100 K to 300 K in 20 ps and then cooled down again to 100 K in 5 ps in the NVT ensemble (canonical ensemble, i.e., constant number of particles N, constant volume V, constant temperature T) with fixed atom positions except for water and ions. In the following step, the system was heated in the NPT ensemble (isothermal-isobaric ensemble, i.e., constant number of particles N, constant pressure P, and constant temperature T) to 300 K over 25 ps without restraining forces on the atoms. Then a 10 ns production trajectory was calculated under periodic boundary conditions, at constant pressure (1 atm) and temperature (300 K) with the weak-coupling algorithm [38] and a time step of 2 fs. The SHAKE [39] algorithm was applied to all bonds involving hydrogen atoms, the particle mesh Ewald method [40] was used to treat the long range electrostatic interactions and a 8.0 Å cut off was applied to van der Waals interactions. Coordinates were saved every ps and energy data every 10th time step. The generated trajectories were all centred to the protein, projected back to their initial solvent boxes and rms fitted to the Cα atom positions of the first frame using the program PTRAJ. Structural descriptors were also calculated using PTRAJ as well as the 2D rmsd values for the 2D rmsd plots. The final plots were created using in-house python scripts and Gnuplot.

### Visualisation

Protein structures were visualised by UCSF Chimera [41]. Data plots were created by GNUPlot (www.gnuplot.info) and python (www.python.org) scripts.

## Results and Discussion

### The Trys Model Containing ADP, Mg^2+^ and a Gsp Analogue

The first TryS model is based on the crystal structure of *Ec*GspS by Pai *et al.* [23] and is meant to explain the first step of T(SH)_2_ biosynthesis (Fig. 1). The model contains ADP, 2 Mg^2+^ ions and a phosphinate analogue of Gsp (see Fig. 2C) known to inhibit GspS as well as TryS [43]. This type of phosphorous-containing pseudopeptides has widely been used to mimic the transition state of peptide bond formation by ATP-dependent ligases [23], [42]–[44] and disclose the approximate position of the γ-phosphate of ATP and, in case of TryS, the productive binding poses of the GSH to be phosphorylated and the Sp to be acylated. Visual inspection of protein conformation snapshots created by molecular dynamics simulation revealed stable conformations and positions of ADP and both Mg^2+^ ions in the respective binding pocket. 2D-rmsd plots of ADP (see Figure S2 in Supporting Information S1 section II) showed one stable conformation over the whole trajectory with only small movements within the binding pocket. In contrast, the Gsp analogue exhibited two major conformations during the simulation (see Figure S2 in Supporting Information S1 section II). In one of the conformations, the spermidine moiety is shifted towards the end of the trajectory (see Figs. 2A and B), whereby the central Sp amine loses the hydrogen bond to Glu 407. This alternate binding mode could explain why the inhibitor is less active against TryS than against GspS [43]. Otherwise the Gsp analogue showed only small movements within the binding site, indicating a reasonable binding mode in the TryS model. Collectively, this data suggests the binding modes of ATP/Mg^2+^, GSH and Sp for Gsp synthesis are essentially the same for *Ec*GspS and *Lm*TryS, as already proposed by Fyfe et al. [19].

**Figure 3:**
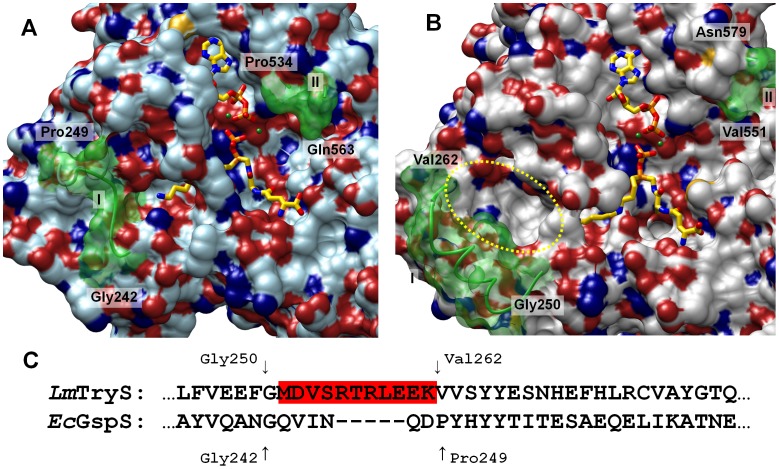
Structure and sequence comparison of the *Ec*GspS crystal structure (pdb: 2ioa; A) and the modeled *Lm*TryS structure (B). Both structures contain ADP, the Gsp analogue (shown as sticks with carbons in yellow) and two Mg^2+^ ions (green balls). The yellow circle highlights an additional pocket in the TryS model which results from sequence differences (C). The green surfaces (I and II) indicate flexible loop regions that were not visible in the *Lm*TryS crystal structure (pdb:2vps): In the *Ec*GspS structure (A) region I (Gly 242 to Pro 249) does not offer any additional binding site near the spermidine moiety of the Gsp analogue, while in the *Lm*TryS model (B) the corresponding loop (Gly250 to 262; marked red in C) builds part of the additional binding pocket. II shows the borders of the ATP Grasp fold loop (Pro534 to Gln563 in *Ec*GspS and Val551to Asn579 in *Lm*TryS, respectively). The loop was removed in both structures to demonstrate the positions of ADP and Mg^2+^.

However, comparison of the *Lm*TryS model (see Fig. 3B) with the *Ec*GspS structure (see Fig. 3A) reveals an interesting difference between the two enzymes that likely explains why only TryS is capable to synthesize T(SH)_2_. In TryS one of the flexible loops that were not visible in the X-ray structure (Fig. 3B: Gly250– Val262) is much longer than in *Ec*GspS (Fig. 3A: Gly242– Pro249). In the TryS model, this loop appears to build an additional pocket close to the site where the spermidine moiety of the Gsp-analogue binds. Sequence alignment reveals that the pocket results from an insertion of 5 residues in the TryS loop region (Fig. 3C). Preliminary docking suggested that the pocket framed by this flexible loop might be the binding site for the glutathionyl moiety of Gsp required for the second step of the catalysis.

**Figure 4:**
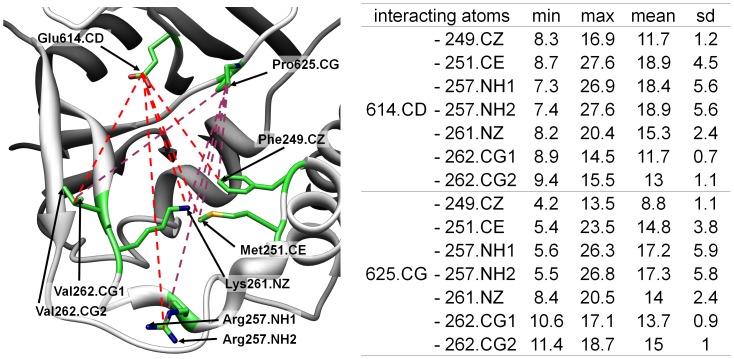
Atomic distances within the ‘new’ binding pocket of *Lm*TryS. Minimum (min) and maximum (max) distances with mean and standard deviations (sd) between Glu614 and Pro626 of the rigid part of the pocket and certain residues of the flexible loop region are given in Å.

Molecular dynamics simulations further supported this possibility. Fig. 4 shows the distances between residues of the flexible loop region and residues of the “rigid” part of the unique TryS pocket. The analysis shows that the pocket stayed open during the whole simulation. The distances between residues of the flexible loop region and Glu614 are in a range between 7.4 Å and 27.6 Å. This is particularly interesting since the loop region proved to be very flexible indeed as expected from the crystal structure (see also Figures S5–S7 and Table S1 in Supporting Information S1 section III). Only the connection between this unique pocket and the Sp binding pocket is closed at some points, the distance between Pro625 and Phe249 having a minimum of 4.2 Å.

**Figure 5:**
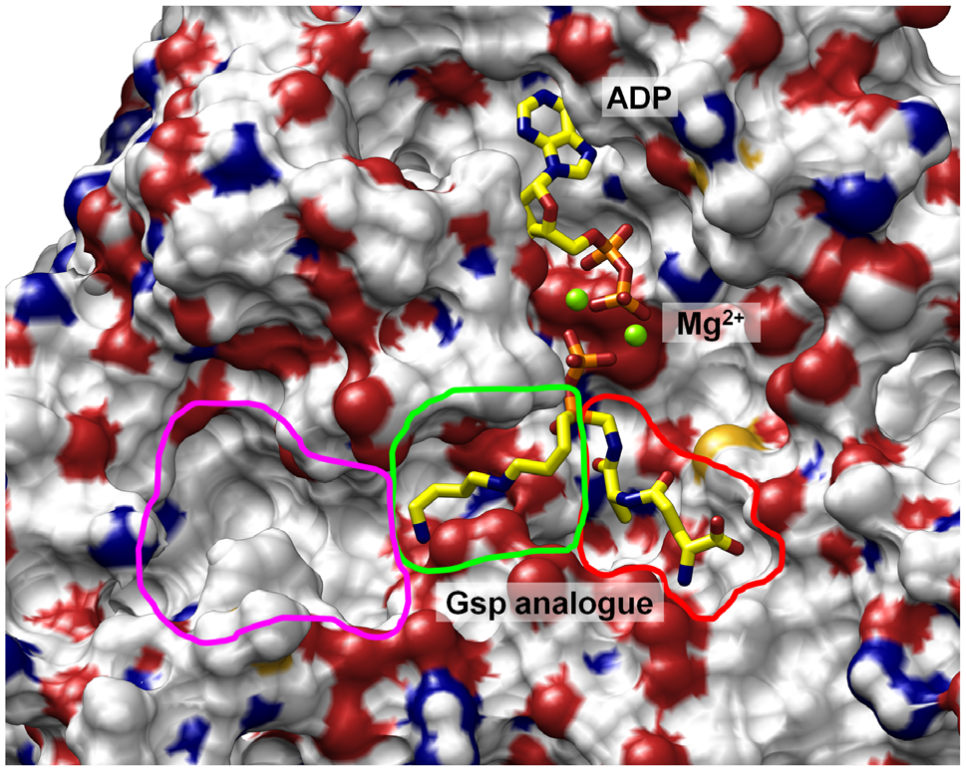
Presumed substrate binding sites of *Lm*TryS as derived from the model with bound ADP/Mg^2+^ and the Gsp analogue. ATP is assumed to essentially occupy the position of ADP. The position of the ATP γ-phosphate is mimicked by the phosphoryl group of the Gsp analogue. The red line marks the binding site of GSH, the green line that of spermidine. The empty pocket surrounded by a magenta line can harber the glutathionyl moiety of Gsp and, together with the spermidine binding site, thus could form a Gsp binding site.

The hypotheses derived from the first model are compiled in Fig. 5. For both steps of trypanothione biosynthesis GSH binds to the same pocket (Fig. 5, red line in lower right corner) in a way that its glycyl carboxyl can interact with both, the γ-phosphate of ATP/Mg^2+^ and an amino group of Sp. The Sp binding region (Fig. 5, green line) is connected to the additional binding pocket (magenta line), presumably destined for accommodating the glutathionyl part of Gsp during the second step of T(SH)_2_ biosynthesis.

### The TryS Models Containing All Substrates

Creating a model with all substrates bound based on the *Lm*TryS crystal structure was not possible. Docking of Gsp simply failed, because Phe 249, which points into the middle of the presumed Gsp pocket (see above), precluded Gsp binding. Therefore, first an *Lm*TryS model containing ATP/Mg^2+^ was generated, as described in Methods. Then, GSH was docked into the binding site evident from the first model and the two-substrate model was subjected to MD simulation. Both substrates retained their positions in their presumed binding site without any major fluctuation, but discrete conformational changes were indicated by the 2D-rmsd and rmsd plots (see Figure S3 in Supporting Information S1). During the simulation, the bond between the ribose and the α-phosphate-group of ATP underwent a conformational change and a flip of the GSH carboxylate near the ATP γ-phosphate was observed.

**Figure 6:**
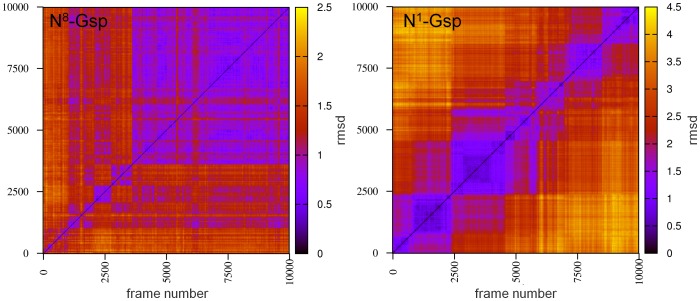
Comparison of N^8^-Gsp and N^1^-Gsp binding to *Lm*TryS by trajectory analysis. 2D-rmsd plots of N^8^-Gsp (left) and N^1^-Gsp (right) are shown for the respective molecular dynamics simulations of the *Lm*TryS model containing all substrates (Gsp, GSH, ATP and two Mg^2+^). Each colour point represents the rmsd between the frame conformation on the x-axis and the frame conformation on the y-axis. The analysis reveals that N^8^-Gsp is essentially moving into a single stable conformation (low rmsd values at the end of the simulation), while N^1^-Gsp adopts several ones.

More importantly, the putative Gsp binding pocket stays open, as in the MD simulation of the first model, and Phe 249, which prevented Gsp docking to the crystal structure (see above), moves out of the binding pocket. In contrast to the *Lm*TryS X-ray structure, representative conformations of this two-substrate model proved to be able to accommodate N^1^-Gsp and N^8^-Gsp at the presumed Gsp pocket. Interestingly, however, docking of the isomers yielded remarkable differences. The docking of N^8^-Gsp creates high-scoring poses that are similar for different protein conformations. In contrast, docking poses for N^1^-Gsp are highly diverse. Moreover, the free amine of Gsp that must interact with the activated GSH is pointing away into the protein in all docking poses, which implies an unfavourable binding mode. Also in MD simulations, the Gsp isomers behave differently. The N^8^-Gsp gradually moves into a stable conformation in its binding pocket (see Fig. 6 left). In contrast, the N^1^-Gsp moves out of the binding pocket without reaching any stable conformation (see Fig. 6 right). Accordingly, N^8^-Gsp appears to be the preferred TryS substrate.

**Figure 7:**
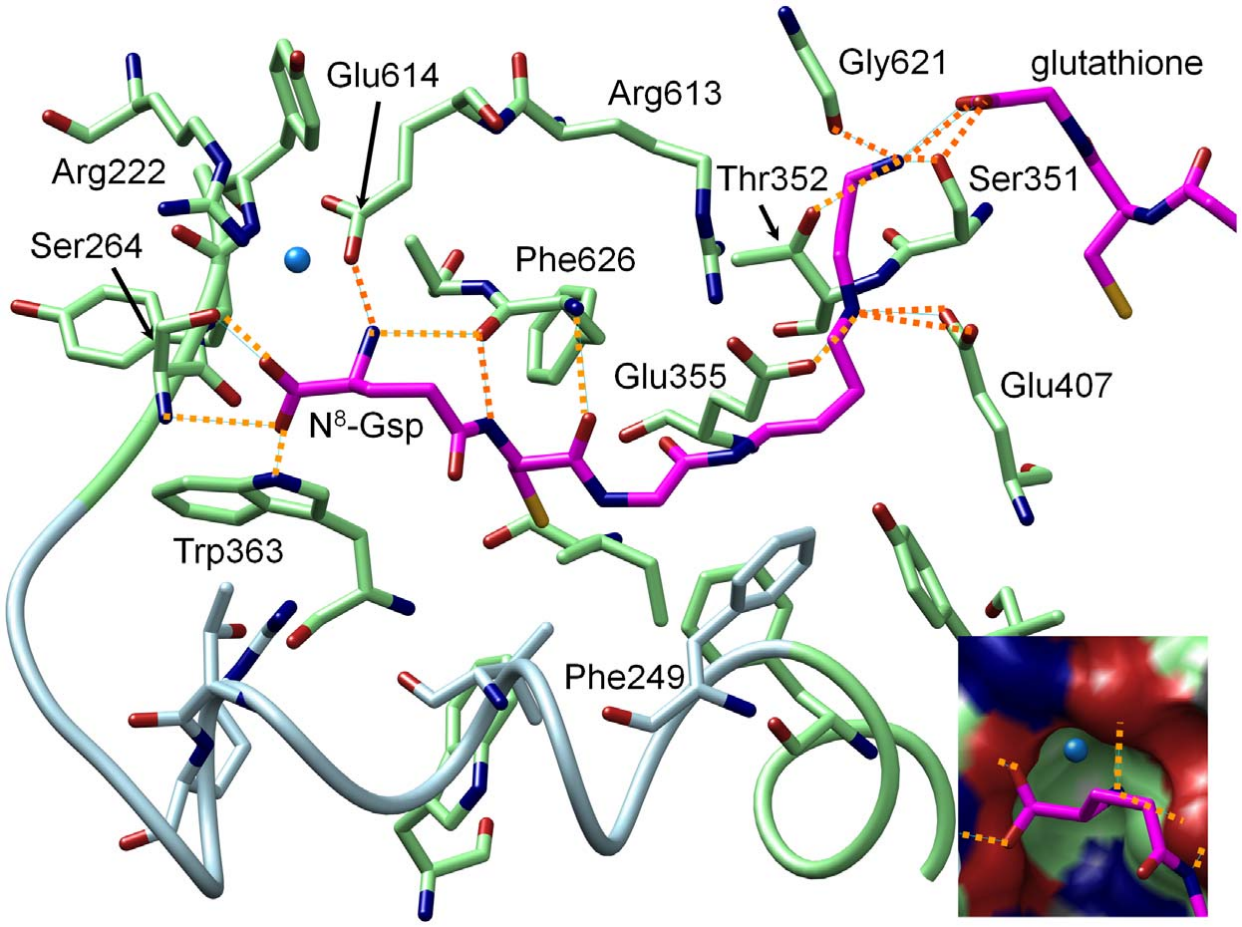
Hydrogen bonding interactions of N^8^-Gsp with protein residues. The inset shows a conserved water molecule in a small pocket near the glutathionyl moiety of Gsp. (magenta: N^8^-Gsp, blue: residues from flexible loop region, green: residues from rigid part of Gsp binding pocket, orange dotted lines: hydrogen bonds). None of the arginine residues directly interacts with the substrate, but both contribute to the stabilisation of the Gsp binding pocket: Arg222 by interacting with Glu614 and Arg613 by interacting with Ser624, Leu274 (not shown) and Glu355.

Figure 7 shows the important hydrogen bonding interactions between N^8^-Gsp and the residues from the Gsp binding site based on the hydrogen bonding analysis (see Figure S14 and Table S2–S3 in Supporting Information S1 section V). The carboxylate terminus interacts with the backbone amide and the hydroxyl group of Ser264 and with Trp363. The adjacent amine interacts with the carboxyl group of Glu614 and the backbone carbonyl group of Phe626. It can be assumed that these residues are functionally important, since all but Ser264 are conserved throughout the species *L. major, L. donovani*, *C. fasciculata*, *T. cruzi* and *T. brucei* (see sequence alignment provided by Comini et al. [16]) and Ser264 is replaced in some sequences by amino acids that can also provide a side chain hydrogen bonding interaction partner (Asn in *C. fasciculata* and Cys in *T. brucei*). Interestingly, a conserved water molecule is observed in a small pocket nearby (Fig. 7) that can also be found in the *Lm*Trys X-ray structure (pdb: 2vob). The amide NH and the carbonyl group of the cysteine residue of Gsp might form hydrogen bonds with the backbone of Phe626. The primary amine of Gsp interacts with the carbonyl group of Gly621, The secondary amine of the spermidine moiety interacts with the carboxy groups of Glu407 and Glu355. Interestingly, the amino acids of the loop region seem to be less important for the binding of Gsp, the additional amino acids in comparison to GspS are only essential to open the pocket for binding. Arg613, which was shown to be essential by Comini et al. [16], shows only a marginal interaction to Gsp. Instead, it interacts via hydrogen bonding interactions with Leu274, Ser624 and Glu355 and can be assumed to be important to stabilize the Sp binding region. Similarly, Arg222 is not directly involved in Gsp binding either, but contributes to the stabilisation of the Gsp binding pocket by interacting with Glu614.

The Gsp binding model here presented complies with earlier reports on T(SH)_2_ biosynthesis. Fairlamb et al. [45] and later Henderson et al. [46] described that the N^8^-Gsp appeared to be the preferred substrate of TryS. This could now be explained by a more stable and productive binding mode of N^8^-Gsp in the Gsp binding pocket. The model further predicts that the Gsp binding pocket could also bind free GSH. If bound this way, GSH would certainly prevent binding of Gsp and likely impair productive binding of Sp, thus explaining the substrate inhibition of TryS by high concentrations of GSH reported earlier [47].

The hydrogen bonding analysis with respect to the substrates ATP/Mg^2+^ and GSH (see Figure S14 and Table S2–S3 in Supporting Information S1 section V) shows that they essentially interact with the protein at the reaction centre as proposed earlier [19]. Asp330 and Glu344 interact and stabilize one Mg^2+^ ion, whereas Glu344 and Asn346 interact with the other Mg^2+^ ion. Both Mg^2+^ ions, Lys513, Lys548 interact with the ATP phosphates. Arg328 can interact with both, the ATP γ-phosphate and the glycyl carboxylate group of GSH at the catalytic centre. This carboxylate is further hydrogen bonded to the OH of Ser351. Its γ-glutamyl carboxylate group sustains hydrogen bonds to Met459, Ser571 and Ser462, the γ-glutamyl amide group shows a permanent hydrogen bond to Glu408 and alternating hydrogen bonds to Asp403 and Thr457. Reliable prediction on further contributions of the ATP grasp loop to substrate binding can not be made, since this loop region did not adopt a stable conformation up to the end of the MD simulation.

**Figure 8:**
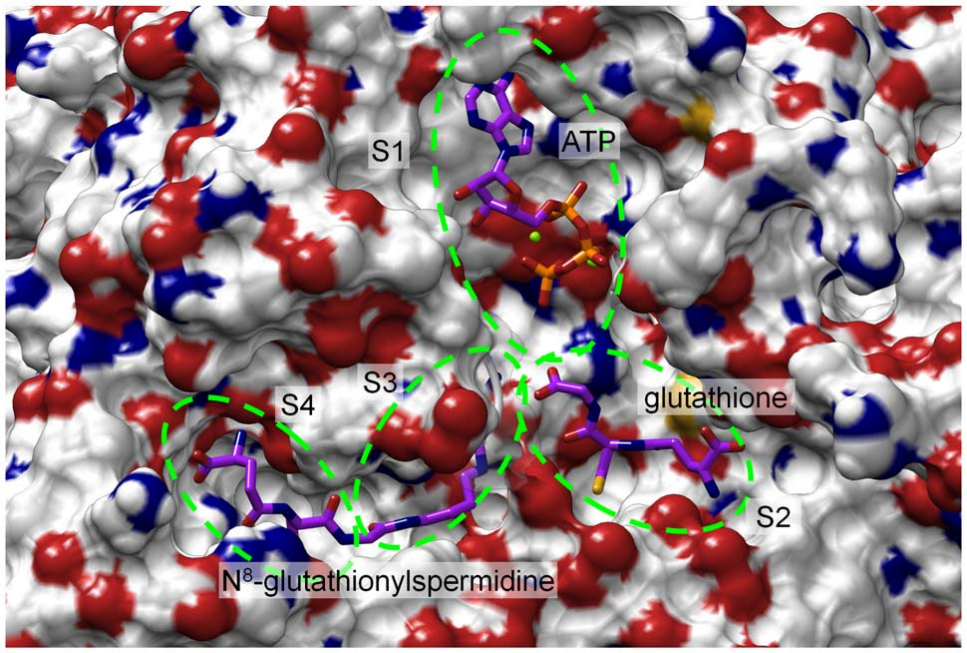
*Lm*TryS model with all substrates. The model represents the last frame of the molecular dynamics simulation including ATP, glutathione and N^8^-Gsp. The substrate binding regions are shown as green circles. S1: ATP binding pocket; S2: glutathione binding pocket, S3: spermidine binding region, S3+S4: N^8^-glutathionylspermidine binding pocket. Substrates are shown with carbon atoms in magenta.

The last frame of the molecular dynamics simulation containing N^8^-Gsp, GSH and ATP (Fig. 8) reveals how the substrates are ideally arranged at the reaction centre for the final step of T(SH)_2_ biosynthesis. The functional groups of the substrates that are involved in the reaction are close to each other. The ATP γ-phosphate points to the carboxyl group of GSH, thus enabling the phosphorylation of its glycine moiety. The amino group of Gsp appears to be localized in an optimum position to accept the glutathionyl residue from the activated GSH. The entire biosynthesis of T(SH)_2_ can now be described as follows: During the first reaction ATP (S1 pocket), GSH (S2 pocket), and spermidine (S3 pocket) bind. After the reaction, the product Gsp, which is generated while bound in the S2 and S3 pockets, has to leave these binding sites to occupy the S3 and S4 pockets in an inverted orientation (see Fig. 8). After binding of the second GSH molecule (S2 pocket) and new ATP (S1 pocket) T(SH)_2_ is then synthesized in an analogous manner.

The sequence of partial reactions is in line with the catalytic model derived from steady state kinetics performed with *Cf*TryS by Comini et al. [16] Based on this analysis a central complex mechanism was postulated for the phosphorylation of GSH by ATP/Mg^2+^, while the partial reactions with Sp or Gsp were best described as ‘ping-pong’ or ‘enzyme substitution’ kinetics, which implies that binding of ATP/Mg^2+^ or GSH occurs independently from that of Sp or Gsp. The initially formed central complex of the enzyme with ATP/Mg^2+^ and GSH or, alternatively, the enzyme with bound glutathionyl-phosphate must be stable and different enough to behave kinetically like a ‘substituted’ enzyme which may react with Sp or Gsp depending on which of the third substrate is bound in the S3 or S3 plus S4 pocket, respectively. In fact, the difference between the substrate-free *Lm*TryS crystal structure [19] and the two- or three-ligand models here described strongly suggests that occupancy of the S1 or S2 pocket modulates the S3/S4 pocket structure for productive binding of Sp or Gsp. Thereby, an enzyme species is generated that, although not substituted in a strict sense, is functionally distinct from the ground state enzyme. Inversely, the decay of the initial complex with liberation of the γ-phosphate of ATP largely depends on the reaction with Sp or Gsp, as is evident from an only marginal ATPase activity of TryS in the absence of the latter substrates [16]. The question which of the catalytic steps triggers the conformational changes that affect substrate binding and allow product release and re-binding of the intermediate Gsp must at present be left unanswered due to still limited structural knowledge.

**Figure 9:**
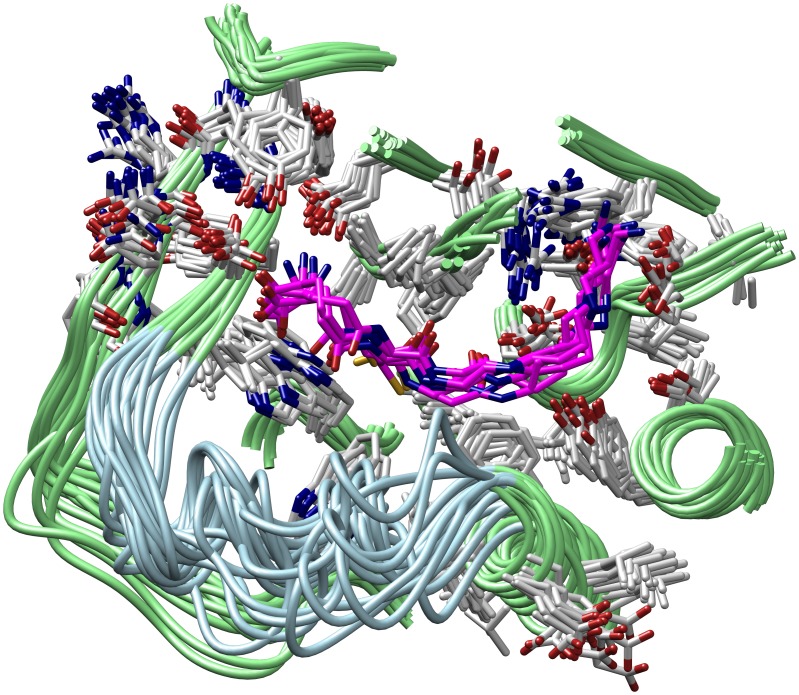
Structural overlay of Gsp binding pocket conformations with bound Gsp. 18 representative conformations are shown (see supporting information II-IV; blue: flexible loop region, green: rigid backbone, grey: rigid residue, magenta: glutathionylspermidine).

**Figure 10:**
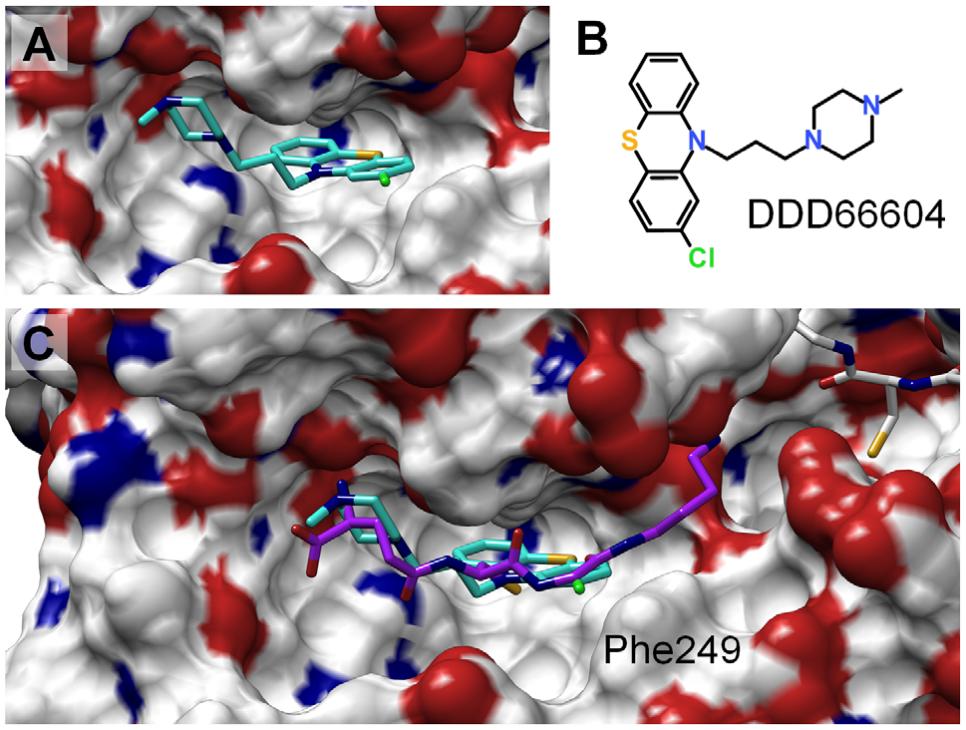
Putative binding mode of TryS inhibitor DDD66604 to *Lm*TryS. A: DDD66604 (turquoise) bound to the Gsp pocket. B: Chemical formula of DDD66604. C: Comparison of binding mode of DDD66604 (turqouise) and N^1^-Gsp (purple).in the Gsp binding pocket.

Instead, we focussed on the more practical question if the generated models might also prove helpful for structure-guided drug design. To this end, a known TryS inhibitor (DDD66604) was docked into representative MD-based conformations. The latter were based on hierarchical clustering of all Gsp binding pocket conformations from all relevant MD simulations (see Figures S4, S5–S7, S8–S10 and S11–S13 in Supporting Information S1 section III). This led to 18 representative conformations of the Gsp pocket (see Fig. 9). The inhibitor (see Fig. 10B) had been described to inhibit TryS with an IC_50_ of 19 µM and to be competed out by spermidine [17], suggesting that the inhibitor binds somewhere in or near the Sp binding region. However, docking of this inhibitor into the original crystal structure again proved to be impossible, since Phe249 blocked the binding position (see previous section). In contrast, docking of the inhibitor using ensemble docking yielded a binding pose that complies with its presumed binding in the Sp pocket (see Fig. 10), which corroborates the usefulness of MD to unravel binding modes. Moreover, the data characterizes the binding pockets of Sp and Gsp, respectively, as structural elements of TryS suitable for structure-based rational drug design.

In conclusion, a computational analysis of *Lm*TryS comprising substrate docking and molecular dynamics substantially complemented the crystallography based understanding of the TryS reaction in providing likely conformations of unresolved protein loops, defining the binding pocket for the intermediate Gsp, and explaining the complex reaction mechanism by re-binding this intermediate from its site of generation to another binding pocket. Moreover, the molecular dynamics-based conformations proved to yield more realistic ligand interaction models than a static X-ray structure, as evidenced by compliance with functional data. It therefore may be expected that the new insight will be of help in the design of novel trypanocidal drugs that are based on specific inhibition of T(SH)_2_ biosynthesis.

## Supporting Information

Supporting Information S1 **This file contains four sections: Section I:** ***Ec*** **GspS and** ***Lm*** **TryS binding site comparison.** Section II: Trajectory Analysis: Ligand rmsd plots. Section III: Trajectory analysis of Gsp binding pocket using hierarchical clustering. Section IV: Hydrogen bonding analysis of final TryS model containing ATP, GSH, N^8^-Gsp and 2 Mg^2+^.
(PDF)
