# Supplementary material for: Molecular Dynamics Reveal Binding Mode of Glutathionylspermidine by Trypanothione Synthetase
Source: PLoS One. 2013 Feb 25;8(2):e56788. doi: 10.1371/journal.pone.0056788 (PMC3581523; doi:10.1371/journal.pone.0056788)
Supplement: Supporting Information S1 — This file contains four sections: Section I: Ec GspS and Lm TryS binding site comparison. Section II: Trajectory Analysis: Ligand rmsd plots. Section III: Trajectory analysis of Gsp binding pocket using hierarchical clustering. Section IV: Hydrogen bonding analysis of final TryS model containing ATP, GSH, N8-Gsp and 2 Mg2+. (PDF) [file pone.0056788.s001.pdf]

<u>**Supporting Information**</u>

I. *Ec*GspS and *Lm*TryS binding site comparison

The binding sites of *Ec*Gsps and *Lm*TryS were overlaid using MOE. Both binding sites are highly conserved regarding sequence and residue conformations.

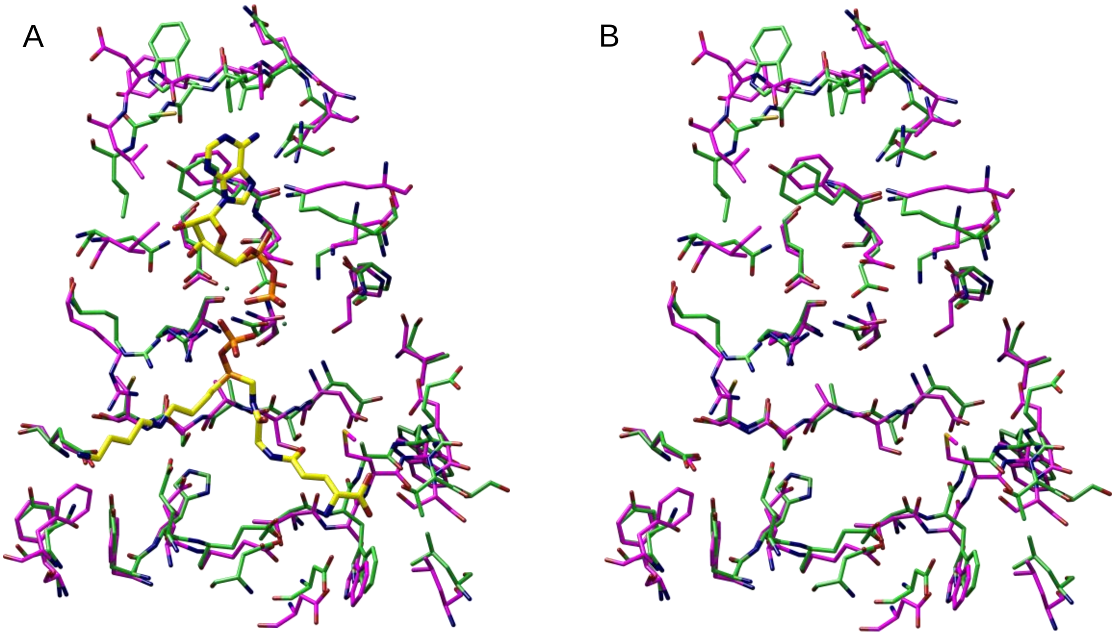

Figure S1: Overlay of *Ec*GspS (pdb: 2ioa) and *Lm*TryS (pdb: 2vps) active site. A: with GspS ligands (ADP, 2 Mg^{2+} ions and the Gsp analogue shown in Fig.2C); B: without ligands. (*Ec*GspS: green: binding site residues; yellow: ligands; *Lm*TryS: magenta: binding site residues)

II. Trajectory Analysis: Ligand rmsd plots

The rmsd plots show the rmsd (root-mean-square-deviation) between the conformation at a specific frame number and the starting conformation at the beginning of the MD simulation (the rmsd value is plotted against the frame number). The 2D rmsd plots show the all-against-all rmsd values between each of the conformations at a specific frame number. Each colour point represents the rmsd between the frame conformation on the x-axis and the frame conformation on the y-axis. Figure S2 shows the 2D-rmsd plots of ADP and the Gsp-analogue from the MD-simulation of the TryS-model containing ADP, Gsp-analogue inhibitor and two Mg2+ ions. Figure S3 shows the analogous analysis of the *Lm*TryS 2-substrate model.

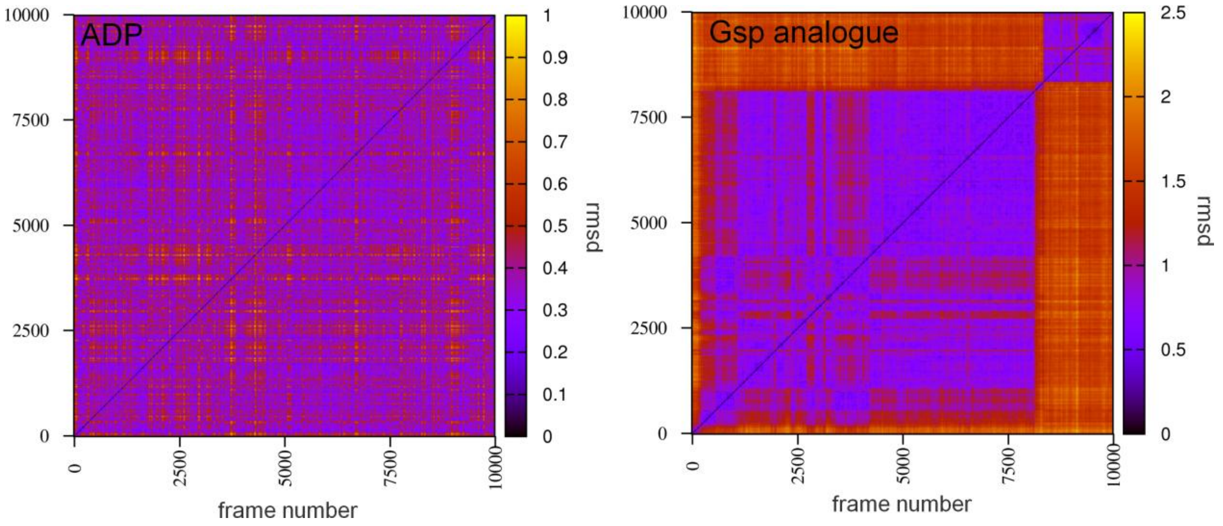

Figure S2: 2D-rmsd plots of ADP (left) and the GSP-analogue (right), based on a fit of the protein conformers onto the C_α-atoms of the binding pocket residues around ADP and the Gsp analogue (taken from the MD-simulation of the TryS-model containing ADP, Gsp-analogue and two Mg^{2+} ions).

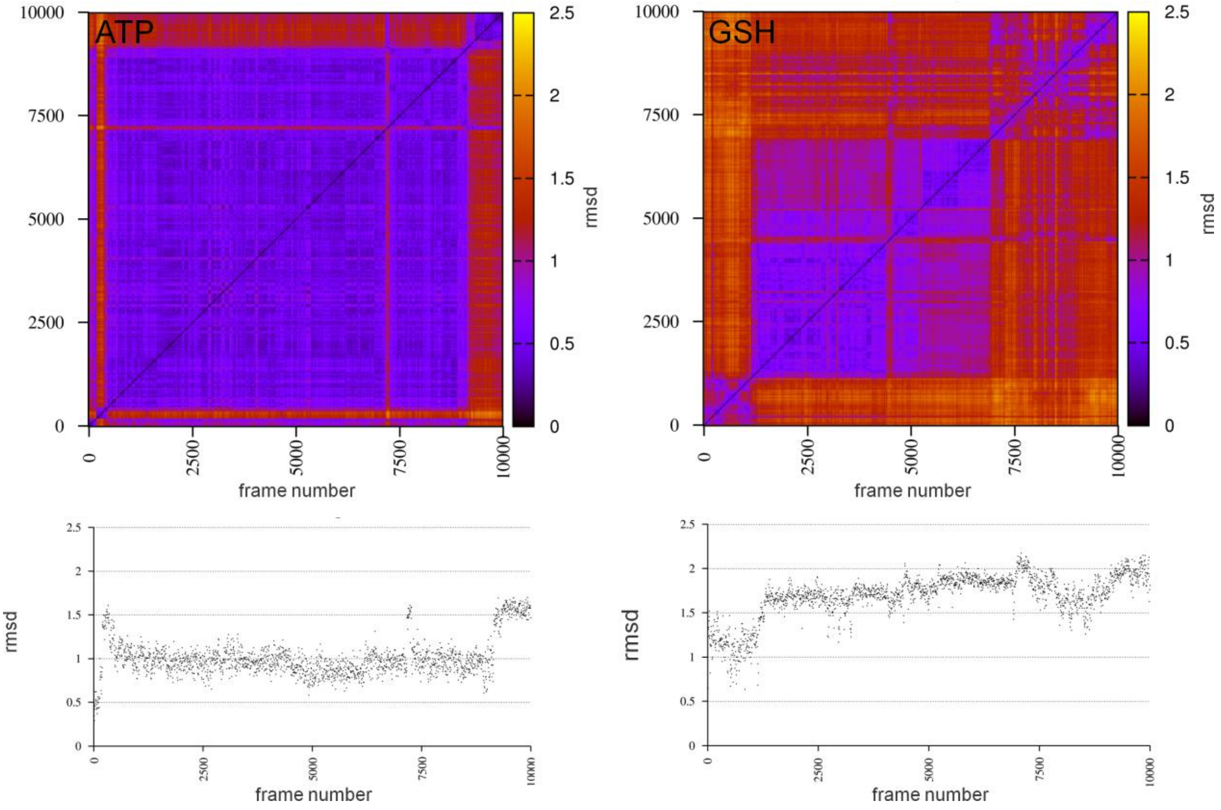

Figure S3: 2D-rmsd (top) and rmsd plots (bottom) of ATP (left) and GSH (right), based on a fit of the protein conformers onto the C_α-atoms of the binding pocket residues around ATP and glutathione (taken from the MD-simulation of the TryS-model containing ATP, GSH and two Mg^{2+} ions). The rmsd plots were calculated based on the initial starting protein conformation of the MD simulation.

III. Trajectory analysis of Gsp binding pocket using hierarchical clustering

For a detailed analysis of the glutathionylspermidine binding pocket a hierarchical clustering of the obtained conformations was performed. As input data the all-against-all rmsd values of all involved residues were used (see legend of Figure S4 for the included residues). These were calculated using PTRAJ. The clustering was done using a hierarchical clustering method in MOE (provided by CCG support) that is based on Ward's Minimum Variance.

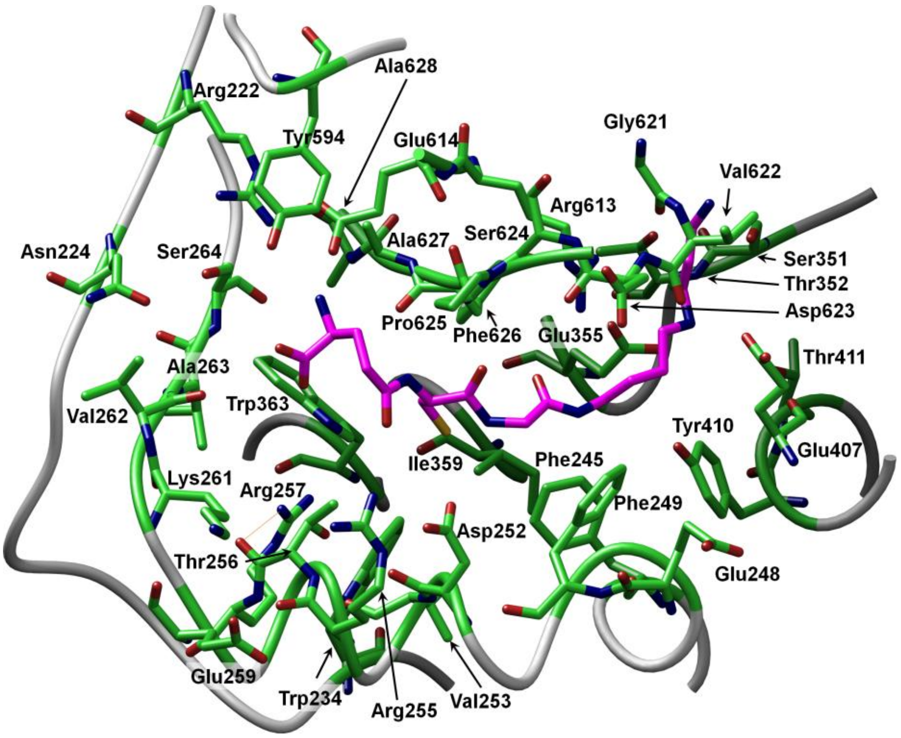

Figure S4: Gsp binding pocket residues that were used for hierarchical clustering of the pocket conformations of the molecular dynamics simulations. (green: pocket residues used, magenta: N^8-Gsp; protein conformation taken from MD simulation including N^8-Gsp).
Used Gsp binding site residues: Arg222, Asn224, Trp234, Phe245, Glu248, Phe249, Asp252, Val253, Arg255, Thr256, Arg257, Glu259, Lys261, Val262, Val263, Ser264, Ser351, Thr352, Glu355, Ile359, Trp363, Glu407, Tyr410, Thr411, Tyr594, Arg613, Glu614, Gly621, Val622, Asp623, Ser624, Pro625, Phe626, Ala627, Ala628

The clustering workflow for all MD simulation started with a 2D rmsd trajectory analysis of the Gsp binding pocket residues (Figure S5, Figure S8 and Figure S11). Afterwards the hierarchical clustering was performed and representative conformations were selected (Figure S6, Figure S9 and Figure S12) which were finally overlaid (Figure S7, Figure S10 and Figure S13).

III.1) TryS-model containing ADP, Gsp-analogue and two Mg^{2+} ions

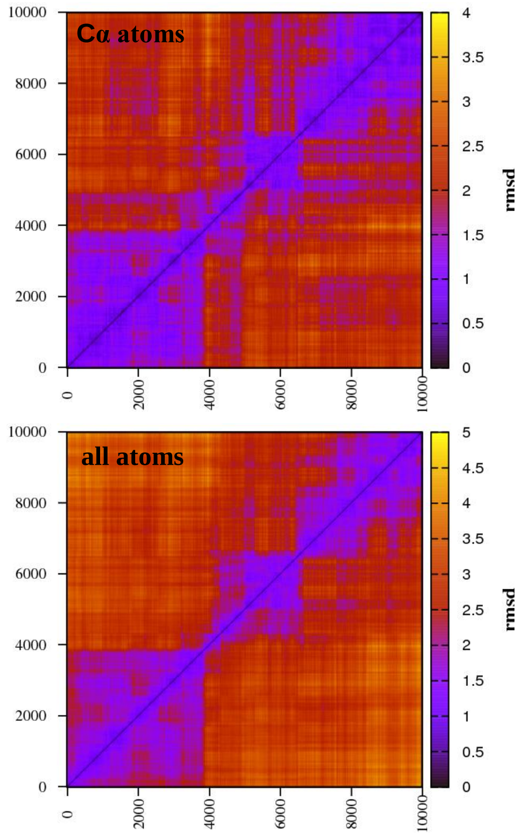

Figure S5: 2D rmsd plots for Gsp binding site residues (top: C_{α}-atoms only, bottom: all atoms). See figure S3 for further information.

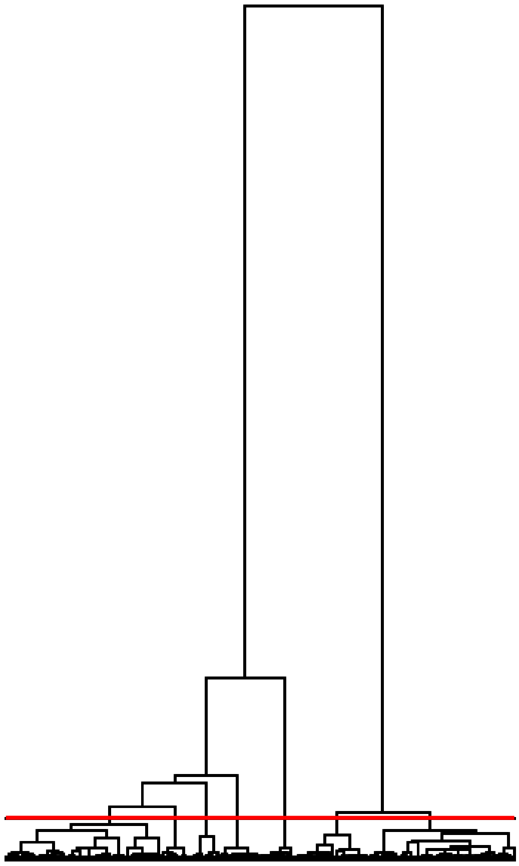

Figure S6: Trajectory hierarchical clustering output based on rmsd values. (red line indicates cluster level: 7 representative conformations).

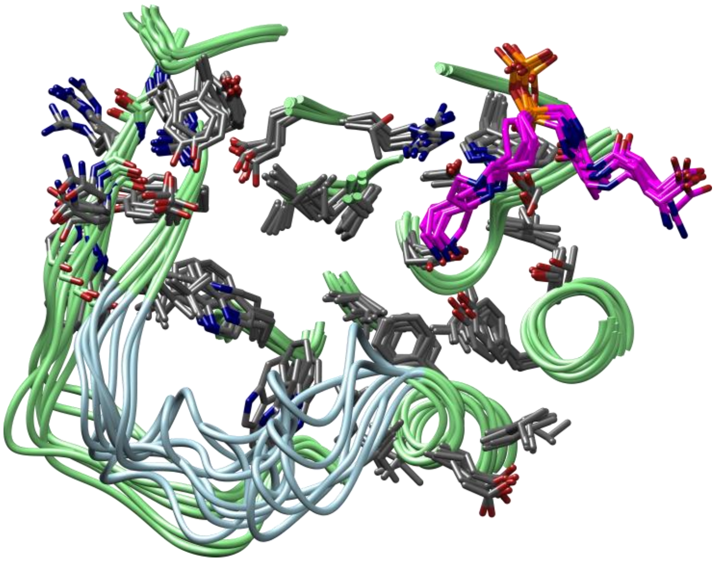

Figure S7: Structural overlay of all 7 representative MD conformations of Gsp binding pocket (blue: flexible loop region; green: rigid backbone; grey: rigid residue; magenta: Gsp analogue).

flexible loop region					
name	min	max	mean	stddev	median
Gly 250	0	0.6	0.3	0.1	0.3
Met 251	0	2.2	1.4	0.3	1.4
Asp 252	0	1.8	0.9	0.4	0.8
Val 253	0	1.6	0.9	0.2	0.9
Ser 254	0	1.5	1.0	0.2	1.0
Arg 255	0	2.4	1.6	0.4	1.6
Thr 256	0	1.9	1.4	0.3	1.5
Arg 257	0	2.3	1.9	0.3	2
Leu 258	0	2.3	1.6	0.5	1.8
Glu 259	0	2.1	1.5	0.3	1.6
Glu 260	0	1.9	1.2	0.4	1.4
Lys 261	0	2.1	1.2	0.5	1.3
Val 262	0	2.1	1.2	0.5	1.1
Val 263	0	2	1.2	0.5	1.1
Ser 264	0	1.6	0.9	0.4	1.1

"rigid" part of Gsp binding pocket					
name	min	max	mean	stddev	median
Ser 351	0	1.5	0.5	0.4	0.4
Thr 352	0	2	1.5	0.2	1.5
Glu 355	0	0.8	0.3	0.1	0.3
Ile 359	0	1.7	1.1	0.4	1.3
Trp 363	0	0.8	0.3	0.1	0.3
Tyr 594	0	1.1	0.6	0.2	0.6
Glu 614	0	1.1	0.8	0.1	0.8
Pro 625	0	1.2	0.8	0.3	1
Phe 626	0	1.2	0.3	0.1	0.3
Ala 627	0	1.1	0.8	0.3	0.9
Ala 628	0	1.1	0.8	0.3	0.9

Table S1: Rmsd values in Å for residues of the Gsp binding pocket with reference to the starting model structure (min: minimum, max: maximun, stddev: standard deviation; measurements include all side chain atoms).

III.2) MD simulation of TryS-model containing ATP, GSH and two Mg^{2+} ions

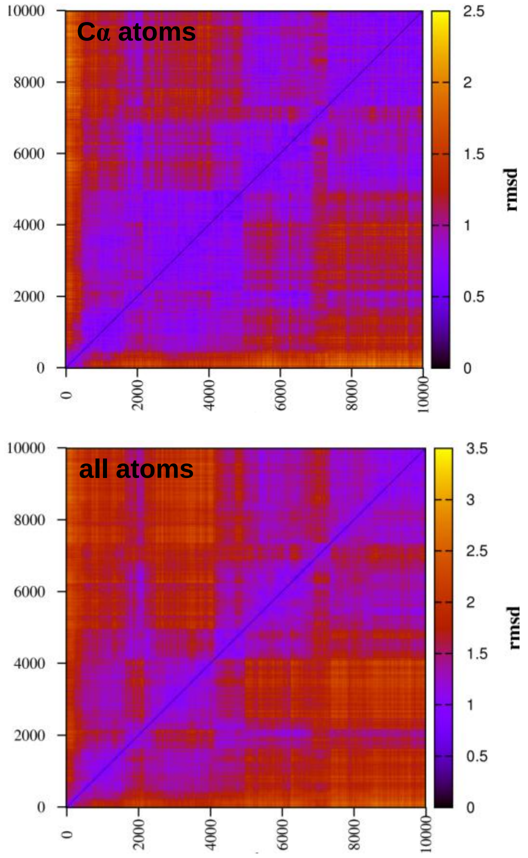

Figure S8: 2D rmsd plots for Gsp binding site residues (top: C_α-atoms only, bottom: all atoms). See figure S3 for further information.

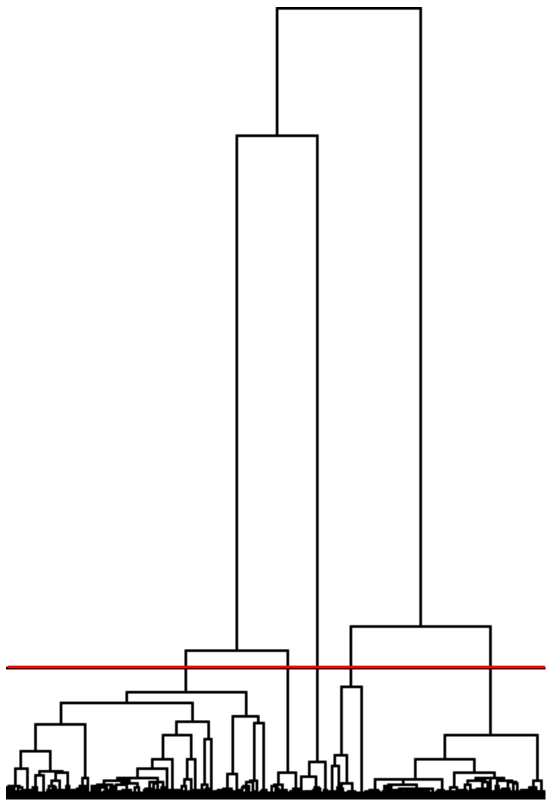

Figure S9: Trajectory hierarchical clustering output based on rmsd values. (red line indicates cluster level: 5 representative conformations).

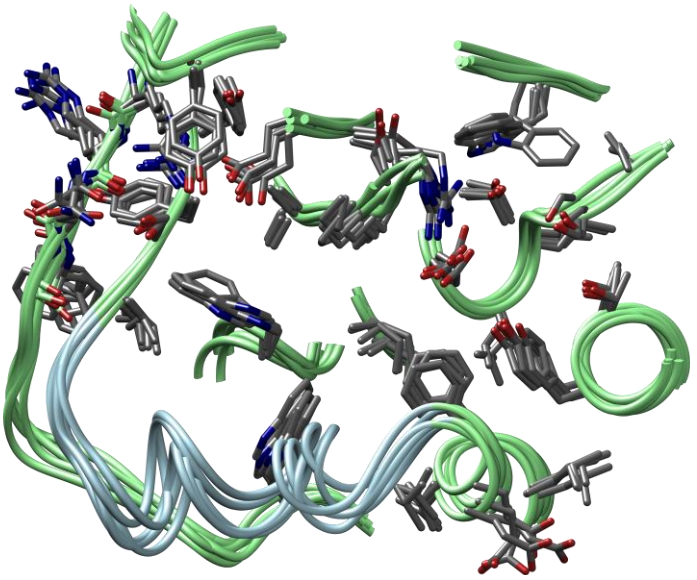

Figure S10: Structural overlay of all 5 representative MD conformations of Gsp binding pocket (blue: flexible loop region; green: rigid backbone; grey: rigid residues)

III.3) MD simulation of TryS-model containing ATP, GSH, N^8-Gsp and two Mg^{2+} ions

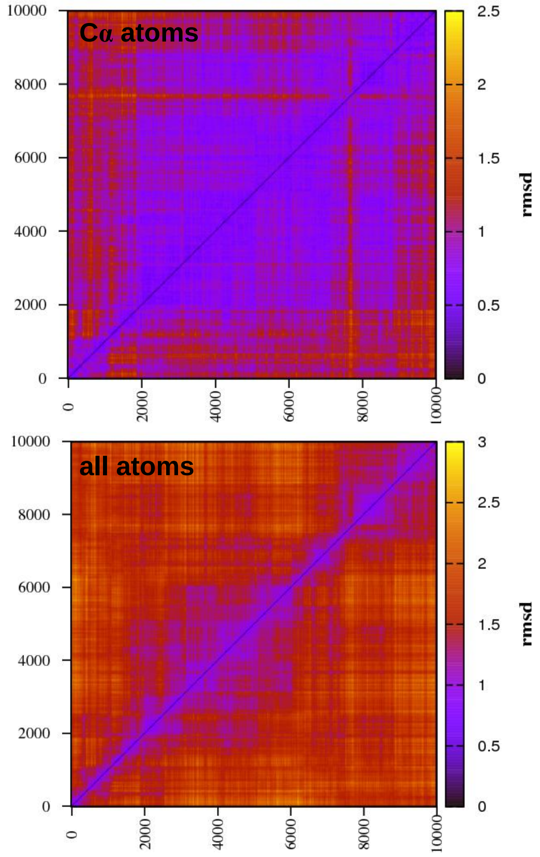

Figure S11: 2D rmsd plots for Gsp binding site residues (top: C_α-atoms only, bottom: all atoms). See figure S3 for further information.

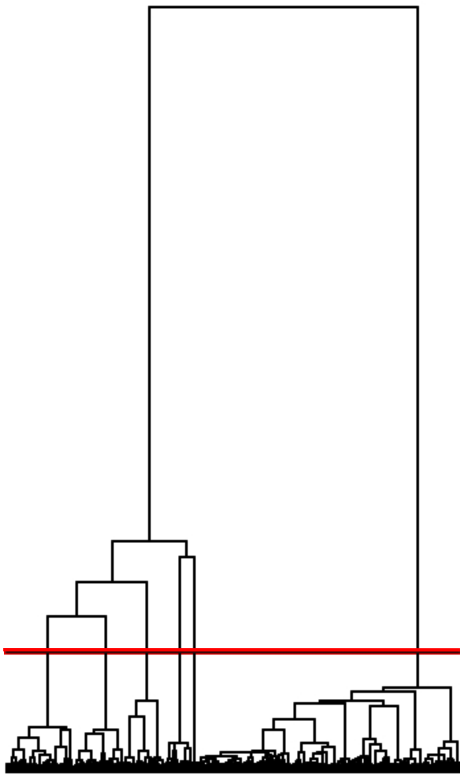

Figure S12: Trajectory hierarchical clustering output based on rmsd values. (red line indicates cluster level: 6 representative conformations)

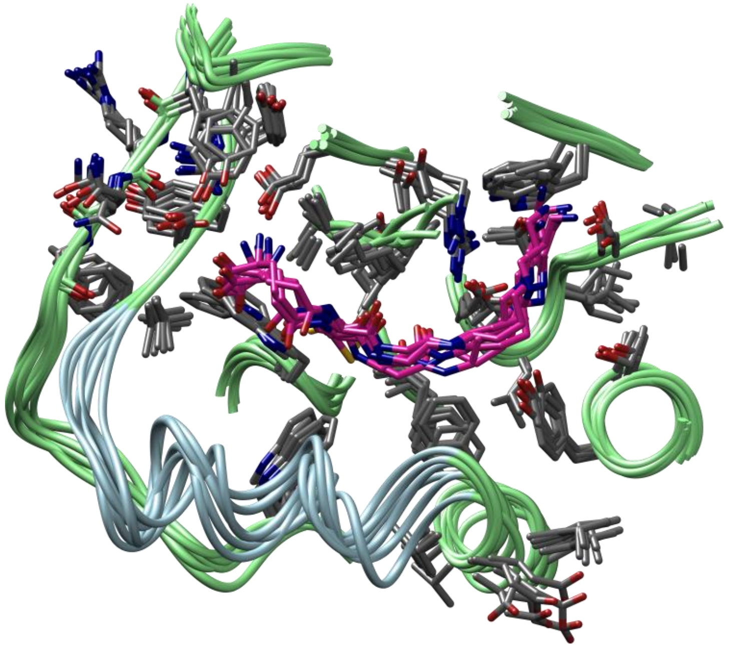

Figure S13: Structural overlay of all 6 representative MD conformations of Gsp binding pocket (blue: flexible loop region; green: rigid backbone; grey: rigid residues; magenta: N^8-Gsp).

IV. Hydrogen bonding analysis of final TryS model containing ATP, GSH, N^8-Gsp and 2 Mg^{2+}

The whole trajectory of the final TryS model was analysed using the PTRAJ H-bond function with a distance cut-off of 3.5Å and an angle cut-off of 60°. Figure S14 shows the atom naming for the substrates. Table S2 shows the results for the ligand acceptor atoms and Table S3 for the ligand donor atoms.

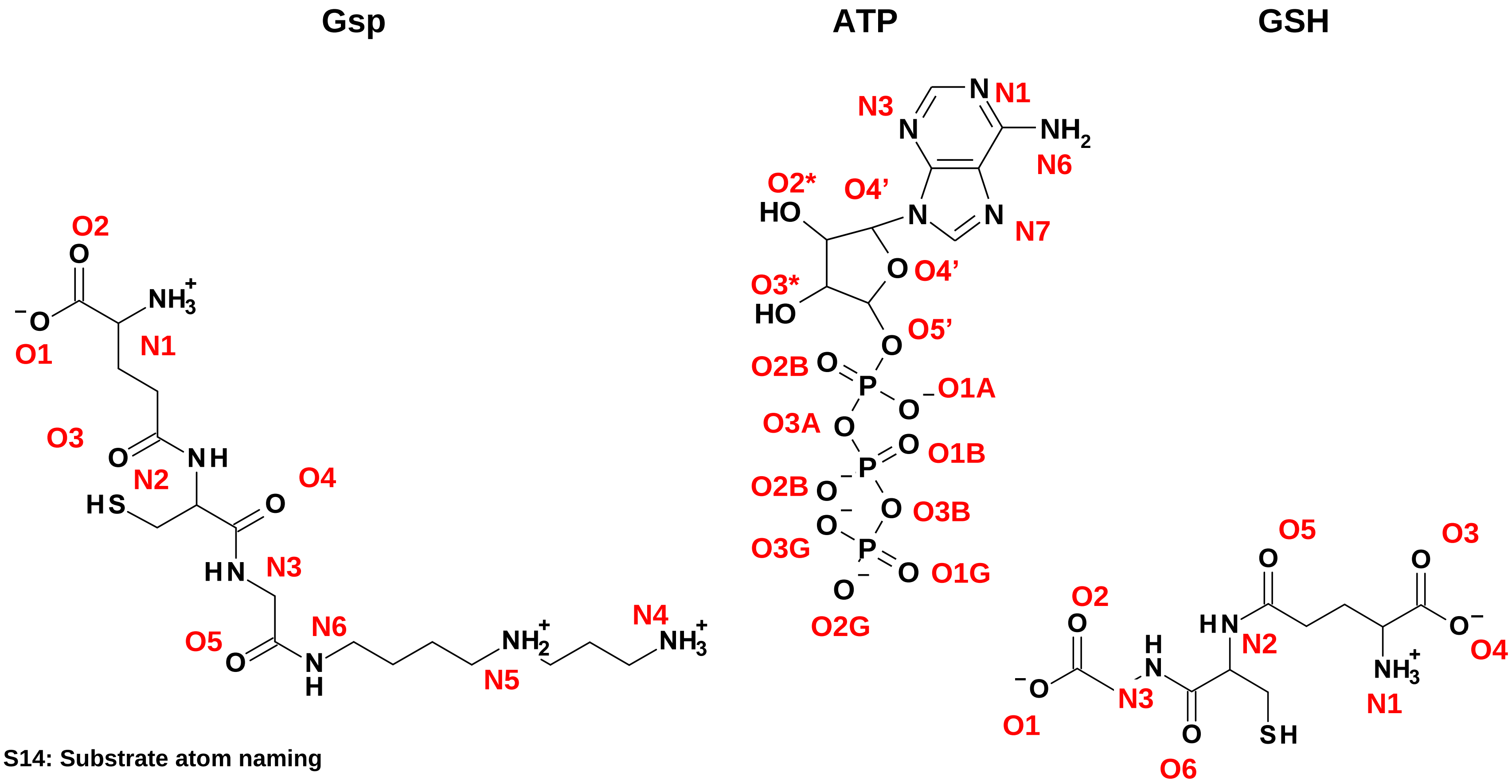

Figure S14: Substrate atom naming

<table>
<tr><th>res@atom</th><th>res@atom</th><th>res@atom</th><th>%occupied</th><th>mean distance</th><th>trajectory hbond occupancy (see Table S2)</th></tr>
<tr><td>GSP@N5</td><td>GSP@H8</td><td>Glu355@OE1</td><td>80.9</td><td>2.811</td><td>|x@@@@@@x@*   .o *@**x@@@*xx@@@@@@@@@@@@@@@@@@@@@|</td></tr>
<tr><td>GSP@N5</td><td>GSP@H8</td><td>Glu355@OE2</td><td>52.31</td><td>3.27</td><td>|o*o*xxxxxo   .- ooo--o-oo-ooxxoxoxxxx*xxxxxxxxxx|</td></tr>
<tr><td>GSP@N5</td><td>GSP@H9</td><td>Glu355@OE1</td><td>10.41</td><td>2.828</td><td>|-*xox. o--   .   ..   . |</td></tr>
<tr><td>GSP@N5</td><td>GSP@H9</td><td>Glu407@OE2</td><td>29.2</td><td>2.868</td><td>|--..-*@o .. .-...   .-*@@@@@@@@- o |</td></tr>
<tr><td>GSP@N5</td><td>GSP@H9</td><td>Glu407@OE1</td><td>28.96</td><td>2.836</td><td>|*@@@xo. *.   .-oo   .*@@@@@@|</td></tr>
<tr><td>GSP@N5</td><td>GSP@H8</td><td>Glu407@OE1</td><td>12.45</td><td>2.844</td><td>|o-oxo   o.   .-o-   -----.-|</td></tr>
<tr><td>GSP@N5</td><td>GSP@H8</td><td>Glu407@OE2</td><td>10.68</td><td>2.877</td><td>| .o-   . .-- ..   ..---o---.-. . |</td></tr>
<tr><td>GSP@N1</td><td>GSP@H2</td><td>Ser264@O</td><td>53.28</td><td>3.048</td><td>|o.-x-x**xx*x-.o@@xo*- .*-..--oox-x*.....o@x..*@@*@|</td></tr>
<tr><td>GSP@N1</td><td>GSP@H3</td><td>Ser264@O</td><td>39.38</td><td>3.039</td><td>|.----.......x-...oo.oxx-*o-*ooooo-.@@@@*..o*o.....|</td></tr>
<tr><td>GSP@N1</td><td>GSP@H1</td><td>Ser264@O</td><td>35.35</td><td>3.044</td><td>|o--oo......-.xo---o-x*@-.*x-xoxx*o-.....o-..o.-.-.|</td></tr>
<tr><td>GSP@N1</td><td>GSP@H3</td><td>Ser264@OG</td><td>12.84</td><td>3.031</td><td>| -ox   -o   o.xoxo*- |</td></tr>
<tr><td>GSP@N1</td><td>GSP@H1</td><td>Ser264@OG</td><td>12.4</td><td>3.019</td><td>| oxo   -- .   -.oooxxo |</td></tr>
<tr><td>GSP@N1</td><td>GSP@H2</td><td>Ser264@OG</td><td>11.25</td><td>3.012</td><td>| oxx   -o .   .--xox.o |</td></tr>
<tr><td>GSP@N1</td><td>GSP@H1</td><td>Glu614@OE1</td><td>48.41</td><td>2.885</td><td>|oox-o*@@@o.-...@@o.@---*x.-x-x--.o* ..-.o@xxo@@-. |</td></tr>
<tr><td>GSP@N1</td><td>GSP@H3</td><td>Glu614@OE1</td><td>42.79</td><td>2.888</td><td>|-oo-xxxxxo.-...xxo.xo**o.*x.oo--oox ....xxo oxo.. |</td></tr>
<tr><td>GSP@N1</td><td>GSP@H2</td><td>Glu614@OE1</td><td>31.16</td><td>2.894</td><td>|-*o-x......-... ....oxx-@xx*oo--o- ..-.-.o@* ... |</td></tr>
<tr><td>GSP@N1</td><td>GSP@H1</td><td>Glu614@OE2</td><td>27.78</td><td>2.94</td><td>| .--.--..o@*x-o. .-   .   ... ..xxxxx. ... x@@|</td></tr>
<tr><td>GSP@N1</td><td>GSP@H2</td><td>Glu614@OE2</td><td>23.05</td><td>2.96</td><td>| .-----... .xx-. .- ...   .- ...@@@*@. ... . |</td></tr>
<tr><td>GSP@N1</td><td>GSP@H3</td><td>Glu614@OE2</td><td>21.58</td><td>2.973</td><td>| .-o---..oxx-x*. .- ---.   -......... ... oxx|</td></tr>
<tr><td>GSP@N1</td><td>GSP@H3</td><td>Phe626@O</td><td>37.7</td><td>3.116</td><td>|o.. -xxxxxo-.-x*o-*-.-x ....--o.ox   o*o .*xxxx|</td></tr>
<tr><td>GSP@N1</td><td>GSP@H1</td><td>Phe626@O</td><td>24.89</td><td>3.124</td><td>|--   ..... -- .--.-. -x..o------.xxxxx..oxo.....|</td></tr>
<tr><td>GSP@N1</td><td>GSP@H2</td><td>Phe626@O</td><td>20.72</td><td>3.096</td><td>|-..   .. ..o-. .-.o** .oo.o---x- .....o ..- |</td></tr>
<tr><td>GSP@N4</td><td>GSP@H6</td><td>Gly621@O</td><td>50.58</td><td>2.889</td><td>|xo-ooxo-o.-.x*. ..-.@@xo-xxoo*x**x*ox..x.-oooxoo*o|</td></tr>
<tr><td>GSP@N4</td><td>GSP@H5</td><td>Gly621@O</td><td>47.14</td><td>2.893</td><td>|xx-*xxx@-.o*o-....o.-.-@x*oox-ox-oo*--.o-*--xo---o|</td></tr>
<tr><td>GSP@N4</td><td>GSP@H7</td><td>Gly621@O</td><td>41.76</td><td>2.895</td><td>|-ooooo*x-.---.....*.oo--x-xxo-oooo-.ox -o-xx-.xx-o|</td></tr>
<tr><td>GSP@N4</td><td>GSP@H7</td><td>GSH@O2</td><td>37.88</td><td>2.817</td><td>|ooooxxo*oox@o--xo.   xoo   oo.o-x@o-xx- oo|</td></tr>
<tr><td>GSP@N4</td><td>GSP@H6</td><td>GSH@O2</td><td>37.74</td><td>2.817</td><td>|oxooxox*ooo-ox*-x-   o-x   .-*o-xoxoooo.--|</td></tr>
<tr><td>GSP@N4</td><td>GSP@H5</td><td>GSH@O2</td><td>36.69</td><td>2.811</td><td>|ooxooxo.oooox*xoo.   -o-   .x-x*.oox-ox.xo|</td></tr>
<tr><td>GSP@N4</td><td>GSP@H5</td><td>GSH@O1</td><td>30.57</td><td>2.826</td><td>|oox--o*xo   .-.-@@x--   -*ooxoo. ..x .   .x |</td></tr>
<tr><td>GSP@N4</td><td>GSP@H7</td><td>GSH@O1</td><td>30.23</td><td>2.826</td><td>|xooo-x--o   .--o-ox@o   ooxxooxo ..x..   .- |</td></tr>
<tr><td>GSP@N4</td><td>GSP@H6</td><td>GSH@O1</td><td>27.5</td><td>2.839</td><td>|oxoo-xx@o   .-**...-x   --oo.o-.   *..   .- |</td></tr>
<tr><td>GSP@N4</td><td>GSP@H5</td><td>Ser351@OG</td><td>13.46</td><td>3.016</td><td>| -xo.-o*-ox *   .   -   ... |</td></tr>
</table>

<table>
<tr><th></th><th></th><th></th><th></th><th></th><th></th></tr>
<tr><td>GSP@N4</td><td>GSP@H6</td><td>Ser351@OG</td><td>13.31</td><td>2.996</td><td>| .   -x--..xxoo x -   -.   -   . |</td></tr>
<tr><td>GSP@N4</td><td>GSP@H7</td><td>Ser351@OG</td><td>11.55</td><td>3.08</td><td>| -x.-o--*-o . -   .   .   .. |</td></tr>
<tr><td>GSP@N4</td><td>GSP@H5</td><td>Thr352@OG1</td><td>7.45</td><td>3.152</td><td>| .-- .oo-o x .   - |</td></tr>
<tr><td>GSP@N4</td><td>GSP@H7</td><td>Thr352@OG1</td><td>6.61</td><td>3.158</td><td>| .o- .o--o x   . |</td></tr>
<tr><td>GSP@N2</td><td>GSP@H15</td><td>Phe626@O</td><td>41.66</td><td>3.202</td><td>|*@x@@xoo--oxooo-oo----.--o-o-oo------.----.-------|</td></tr>
<tr><td>GSH@N1</td><td>GSH@H8</td><td>Glu408@OE1</td><td>62.82</td><td>2.917</td><td>|-**@xo*x-x@o**x.-@@x@xo..x*xo*xo.****.-**x.xxxx-- |</td></tr>
<tr><td>GSH@N1</td><td>GSH@H6</td><td>Glu408@OE2</td><td>62.29</td><td>2.966</td><td>|-*x**@***oxo*@x .xx*xoo xx*ox*o oxxx .xxx.@o@@@o-|</td></tr>
<tr><td>GSH@N1</td><td>GSH@H8</td><td>Glu408@OE2</td><td>58.04</td><td>2.973</td><td>|xx@@o xo-*@-. .ox@@o*xx**xx--@.*x*xxxxoxxoo o --oo|</td></tr>
<tr><td>GSH@N1</td><td>GSH@H7</td><td>Glu408@OE1</td><td>53.83</td><td>2.922</td><td>|x-o------ooo..-@*xxo*xx@@ox-xx-x@xoxo@*oxo*.x.. ..|</td></tr>
<tr><td>GSH@N1</td><td>GSH@H6</td><td>Glu408@OE1</td><td>53.32</td><td>2.929</td><td>|x--.x@oo*-.**@@*x .x.oo---ox*-*ox----xx-oxx@o@x--.|</td></tr>
<tr><td>GSH@N1</td><td>GSH@H7</td><td>Glu408@OE2</td><td>48.46</td><td>2.966</td><td>|@.. o*--x- xooo@* o.oo@@--o*.oo@...-@*..o*xoxo.x@|</td></tr>
<tr><td>GSH@N1</td><td>GSH@H6</td><td>ASP403@OD2</td><td>16.87</td><td>2.949</td><td>|ox**xx**xx- .   ---. |</td></tr>
<tr><td>GSH@N1</td><td>GSH@H8</td><td>ASP403@OD2</td><td>15.79</td><td>2.95</td><td>|*oxxo.o--x-   ..   o@x. |</td></tr>
<tr><td>GSH@N1</td><td>GSH@H7</td><td>ASP403@OD2</td><td>14.04</td><td>2.956</td><td>|x---o*-o*-. . ..   -xo. |</td></tr>
<tr><td>GSH@N1</td><td>GSH@H8</td><td>Thr457@OG1</td><td>10.09</td><td>3.289</td><td>| .-... ...--.-   ....-- .- . ..-. |</td></tr>
<tr><td>GSH@N1</td><td>GSH@H7</td><td>Thr457@OG1</td><td>8.51</td><td>3.299</td><td>| ..-   --...-..   ....--... .. . |</td></tr>
<tr><td>GSH@N1</td><td>GSH@H6</td><td>Thr457@OG1</td><td>8.02</td><td>3.289</td><td>| -...-. - -.   .- . . .. -. |</td></tr>
<tr><td>ATP@N6</td><td>ATP@H61</td><td>Gln584@O</td><td>99.51</td><td>2.94</td><td>|@@|</td></tr>
<tr><td>ATP@N6</td><td>ATP@H60</td><td>Gln583@OE1</td><td>89.2</td><td>3.078</td><td>|*********xx**************x*xx*@******@@@@@@@@***@@|</td></tr>
<tr><td>ATP@N6</td><td>ATP@H60</td><td>Gln584@O</td><td>7.59</td><td>2.978</td><td>|.. .   .   .   .|</td></tr>
<tr><td>ATP@O3*</td><td>ATP@H3'</td><td>Asp330@OD1</td><td>87.14</td><td>2.863</td><td>|-@@*@@@*x*****x**@@*@*@*@@@@@@@@@@@xo***oo*x******|</td></tr>
</table>

Table S2: Hydrogen bonding analysis using PTRAJ H-bond function for ligand (N^8-GSP, GSH, ATP) hydrogen bonding donor atoms (heavy atom + hydrogen) interacting with residue acceptor atoms. The trajectory H-bond occupancy describes the percentage of formed hydrogen bonds during a specific time series step along the trajectory (0-5%: ‘ ‘, 5-20%:‘.‘, 20-40%: ‘-‘, 40-60%:‘o‘, 60-80%:‘x‘, 80-95%:‘*‘, 95-100%:‘@‘)

<table>
<tr><th>res@atom</th><th>res@atom</th><th>res@atom</th><th>%occupied</th><th>distance</th><th>trajectory hbond occupancy</th></tr>
<tr><td>GSP@O4</td><td>Phe626@H</td><td>Phe626@N</td><td>86.04</td><td>2.998</td><td>|@.xx-.ox--*@@@**@*@*@@@@@@@@@@@@@@****@@@@@@@@@**@@|</td></tr>
<tr><td>GSP@O1</td><td>Ser264@H</td><td>Ser264@N</td><td>78.09</td><td>3.045</td><td>|xxo-x*@@@@*x*@*@*ox******x*xooo.oox*******x*x*****|</td></tr>
<tr><td>GSP@O1</td><td>Ser264@HG</td><td>Ser264@OG</td><td>5.63</td><td>2.931</td><td>| -x .*o. |</td></tr>
<tr><td>GSP@O1</td><td>Trp363@HE1</td><td>Trp363@NE1</td><td>10.09</td><td>3.019</td><td>|. -.. oxo*o-. . . . |</td></tr>
<tr><td>GSP@O2</td><td>Ser264@H</td><td>Ser264@N</td><td>73.47</td><td>3.109</td><td>|.-o-oxxoxxxxxxxx**xxx****xxx**x*x**xxxx*x**x**x***|</td></tr>
<tr><td>GSP@O2</td><td>Ser264@HG</td><td>Ser264@OG</td><td>45.5</td><td>2.948</td><td>| -ox-xoxooooxoxo-oo--ooo. -o-.-oooxooxoooooxooo*|</td></tr>
<tr><td>GSP@O5</td><td>Arg613@HH22</td><td>Arg613@NH2</td><td>9.03</td><td>3.268</td><td>| ...-. -.... . .. .-.-.|</td></tr>
<tr><td>GSP@O5</td><td>Arg613@HH21</td><td>Arg613@NH2</td><td>8.92</td><td>3.268</td><td>| ...-. -.... . .. .-.-.|</td></tr>
<tr><td>GSH@O5</td><td>Ser349@HG</td><td>Ser349@OG</td><td>80.48</td><td>2.852</td><td>| ..oxo.-oo*@@@@@@@@**@x--@@@@@@@@@@@@@@*x*@@*@@@@@|</td></tr>
<tr><td>GSH@O6</td><td>Ser351@HG</td><td>Ser351@OG</td><td>76.92</td><td>2.874</td><td>|.. -xxoo*@@@@@@@*@*@@@@*@o-*x-o**x**@**@*@***xxxo.|</td></tr>
<tr><td>GSH@O6</td><td>Arg328@HH21</td><td>Arg328@NH2</td><td>9.27</td><td>3</td><td>| -@@x -- . @ . |</td></tr>
<tr><td>GSH@O6</td><td>Arg328@HH11</td><td>Arg328@NH1</td><td>9.24</td><td>3.046</td><td>|x*x*@o |</td></tr>
<tr><td>GSH@O6</td><td>Arg328@HH12</td><td>Arg328@NH1</td><td>6.88</td><td>3.042</td><td>|xooxo- |</td></tr>
<tr><td>GSH@O1</td><td>Arg328@HH21</td><td>Arg328@NH2</td><td>46.64</td><td>2.81</td><td>|. -*@@@@@@@- @@*. x*@*@ **@***.**|</td></tr>
<tr><td>GSH@O1</td><td>Arg328@HE</td><td>Arg328@NE</td><td>28.55</td><td>3.093</td><td>| .. --oxo--*. *xo- . ox*oo -o*oox.xx|</td></tr>
<tr><td>GSH@O1</td><td>Ser351@HG</td><td>Ser351@OG</td><td>40.07</td><td>2.989</td><td>|*@@o o -..---..--oo-o---. -x.o**@*ooo-o .-o--ox--|</td></tr>
<tr><td>GSH@O1</td><td>GSP@H5</td><td>GSP@N4</td><td>30.57</td><td>2.826</td><td>|oox--o*xo .-.-@@x-- -*ooxoo. ..x . .x |</td></tr>
<tr><td>GSH@O1</td><td>GSP@H7</td><td>GSP@N4</td><td>30.23</td><td>2.826</td><td>|xooo-x--o .--o-ox@o ooxxooxo ..x.. .- |</td></tr>
<tr><td>GSH@O1</td><td>GSP@H6</td><td>GSP@N4</td><td>27.5</td><td>2.839</td><td>|oxoo-xx@o .-**...-x --oo.o-. *.. .- |</td></tr>
<tr><td>GSH@O2</td><td>Ser351@HG</td><td>Ser351@OG</td><td>38.28</td><td>3.013</td><td>|- o-o -xoooo-o---..... xx-.-.-o--x*xo.---***xoo.|</td></tr>
<tr><td>GSH@O2</td><td>GSP@H5</td><td>GSP@N4</td><td>36.69</td><td>2.811</td><td>|ooxooxo.oooox*xoo. -o- .x-x*.oox-ox.xo|</td></tr>
<tr><td>GSH@O2</td><td>GSP@H7</td><td>GSP@N4</td><td>37.88</td><td>2.817</td><td>|ooooxxo*oox@o--xo. xoo oo.o-x@o-xx- oo|</td></tr>
<tr><td>GSH@O2</td><td>GSP@H6</td><td>GSP@N4</td><td>37.74</td><td>2.817</td><td>|oxooxox*ooo-ox*-x- o-x .-*o-xoxoooo.--|</td></tr>
<tr><td>GSH@O2</td><td>Arg328@HH21</td><td>Arg328@NH2</td><td>31.92</td><td>2.811</td><td>| . x*@@@@xx ..@@@@@@@- . .* |</td></tr>
<tr><td>GSH@O2</td><td>Arg328@HE</td><td>Arg328@NE</td><td>20.14</td><td>3.07</td><td>| . -o-oo-o. - xxxx***- x |</td></tr>
<tr><td>GSH@O4</td><td>Met459@H</td><td>Met459@N</td><td>37.06</td><td>3.072</td><td>| -@@@@*@@@*-o .*@@x .*@**x. |</td></tr>
<tr><td>GSH@O4</td><td>Ser571@HG</td><td>Ser571@OG</td><td>19.2</td><td>2.918</td><td>| --**- .*x -x.*@*. .-x|</td></tr>
<tr><td>GSH@O4</td><td>Ser462@HG</td><td>Ser462@OG</td><td>8.13</td><td>3.031</td><td>| - . - -. -*x o. |</td></tr>
<tr><td>GSH@O3</td><td>Ser571@HG</td><td>Ser571@OG</td><td>32.3</td><td>2.796</td><td>| ..x- ***@ox*@@o . o **@@*o@|</td></tr>
<tr><td>GSH@O3</td><td>Met459@H</td><td>Met459@N</td><td>26.17</td><td>3.041</td><td>| oo o@@*@*@@@*@@x |</td></tr>
<tr><td>GSP@O3</td><td>Trp363@HE1</td><td>Trp363@NE1</td><td>18.01</td><td>3.19</td><td>| ... .o-.-.. .-ooxooo---o-o---|</td></tr>
<tr><td>GSH@O3</td><td>Ser462@HG</td><td>Ser462@OG</td><td>8.38</td><td>3.013</td><td>| - . - o. -@x o. |</td></tr>
<tr><td>ATP@N7</td><td>Lys548@HZ1</td><td>Lys548@NZ</td><td>91.38</td><td>2.955</td><td>|x---o*@**@*@@-x@@@@@@@@@@@@@@@@@@@@@@@@@@@@@@@@@@@|</td></tr>
</table>

<table>
<tr><td>ATP@N7</td><td>Lys548@HZ3</td><td>Lys548@NZ</td><td>83.29</td><td>2.955</td><td>|o@@@*-o---o--@***x*****@@*@****@@@@*@******@@@@@**|</td></tr>
<tr><td>ATP@N7</td><td>Lys548@HZ2</td><td>Lys548@NZ</td><td>58.43</td><td>2.97</td><td>|@@**@@@@@@@@@@*ooooo--o-------------o-ooo-oo-oo@*oo|</td></tr>
<tr><td>ATP@N1</td><td>Phe586@H</td><td>Phe586@N</td><td>89.87</td><td>3.166</td><td>|x*@*@**@*@*@***@@**********x*xo**xx*@***@@@***@*@*|</td></tr>
<tr><td>ATP@O1A</td><td>Lys548@HZ2</td><td>Lys548@NZ</td><td>79.95</td><td>2.755</td><td>|-xoxo........o*@@@@@@@@@@@@@@@@@@@@@@@@@@@@@@@@@@@|</td></tr>
<tr><td>ATP@O1A</td><td>Lys548@HZ3</td><td>Lys548@NZ</td><td>61.71</td><td>2.754</td><td>|x.-.o@@@@@@@@.-ooo--ooxoooooxxxxxxxoxxooooxxxx*xxx|</td></tr>
<tr><td>ATP@O1A</td><td>Lys548@HZ1</td><td>Lys548@NZ</td><td>31.07</td><td>2.757</td><td>|x@@@*oooooooox@o-----............. ....|</td></tr>
<tr><td>ATP@O3G</td><td>Asn346@HD21</td><td>Asn346@ND2</td><td>54.82</td><td>3.296</td><td>|*@@@@*xoxxxxxoxxxxooooxooxxxxooo----oo------------|</td></tr>
<tr><td>ATP@O3G</td><td>Arg328@HH21</td><td>Arg328@NH2</td><td>10.1</td><td>2.935</td><td>|o@@@*o |</td></tr>
<tr><td>ATP@O3G</td><td>Arg328@HH22</td><td>Arg328@NH2</td><td>5.01</td><td>2.989</td><td>|---ox- |</td></tr>
<tr><td>ATP@O2B</td><td>Lys513@HZ2</td><td>Lys513@NZ</td><td>24.27</td><td>2.911</td><td>|o   . oo*.*-o*x.-o****xxx|</td></tr>
<tr><td>ATP@O2B</td><td>Lys513@HZ3</td><td>Lys513@NZ</td><td>23.54</td><td>2.92</td><td>|-   o*@.x-x-o@xx*o.o.o@o|</td></tr>
<tr><td>ATP@O2B</td><td>Lys513@HZ1</td><td>Lys513@NZ</td><td>20.78</td><td>2.931</td><td>|.   .xo.o@oo*o.*x-ooooo.-|</td></tr>
<tr><td>ATP@O1G</td><td>Arg328@HH21</td><td>Arg328@NH2</td><td>10.88</td><td>3.014</td><td>|@**@@x |</td></tr>
<tr><td>ATP@O1G</td><td>Arg328@HE</td><td>Arg328@NE</td><td>5.11</td><td>3.207</td><td>|-oxo-- |</td></tr>
<tr><td>ATP@O3A</td><td>Lys513@HZ2</td><td>Lys513@NZ</td><td>6.1</td><td>3.33</td><td>| ... -..-.......---.|</td></tr>
<tr><td>ATP@O3A</td><td>Lys513@HZ1</td><td>Lys513@NZ</td><td>5.41</td><td>3.325</td><td>| ..-.-. ......--..|</td></tr>
<tr><td>ATP@O3A</td><td>Lys513@HZ3</td><td>Lys513@NZ</td><td>5.26</td><td>3.337</td><td>| .......-...-.. .--.|</td></tr>
</table>

Table S3: Hydrogen bonding analysis using PTRAJ H-bond function for ligand (N^8-GSP, GSH, ATP) hydrogen bonding acceptor atoms interacting with residue donor atoms (heavy atom + hydrogen). The trajectory H-bond occupancy describes the percentage of formed hydrogen bonds during a specific time series step along the trajectory (0-5%: ' ', 5-20%:'.', 20-40%: '-', 40-60%:'o', 60-80%:'x', 80-95%:'*', 95-100%:'@')
